# Exosome and lipid metabolism-related genes in pancreatic adenocarcinoma: a prognosis analysis

**DOI:** 10.18632/aging.205130

**Published:** 2023-10-18

**Authors:** Jia Wu, Yajun Li, Ghulam Nabi, Xin Huang, Xu Zhang, Yuanzhen Wang, Liya Huang

**Affiliations:** 1 Department of Gastroenterology, General Hospital of Ningxia Medical University, Yinchuan, Ningxia, China; 2 Institute of Nature Conservation, Polish Academy of Sciences, Krakow, Poland; 3 Department of Gastroenterology, Traditional Chinese Medicine Hospital of Yinchuan, Yinchuan, Ningxia, China

**Keywords:** pancreatic adenocarcinoma, prognosis analysis, exosome, lipid metabolism

## Abstract

Objective: The purpose of the study was to investigate the role of exosome and lipid metabolism-related genes (EALMRGs) mRNA levels in the diagnosis and prognosis of Pancreatic Adenocarcinoma (PAAD).

Methods: The mRNA expression pattern of PAAD and pan-cancers with prognostic data were obtained from The Cancer Genome Atlas (TCGA) and Gene Expression Omnibus (GEO) database. EALMRGs were acquired from GeneCards and MSigDB database after merging and deduplication. Prognostic EALMRGs were screened through univariate COX regression analysis, and a prognostic model was constructed based on these genes by least absolute shrinkage and selection operator (LASSO) regression. The prognostic value of EALMRGs was then validated in pan-cancer data. The time characteristics ROC curve analysis was performed to evaluate the effectiveness of the prognostic genes.

Results: We identified 5 hub genes (ABCB1, CAP1, EGFR, PPARG, SNCA) according to high and low-risk groups of prognoses. The risk formula was verified in three other cohort of pancreatic cancer patients and was explored in pan-cancer data. Additionally, T cell and dendritic cell infiltration was significantly increased in low-risk group. The expression of the 5 hub genes was also identified in single-cell sequencing data of pancreatic cancer with pivotal pathways. Additionally, functional enrichment analysis based on pancreatic cancer data in pancreatic cancer showed that protein serine/threonine kinase activity, focal adhesion, actin binding, cell-substrate junction, organic acid transport, and regulation of transporter activity were significant related to the expression of genes in EALMRGs.

Conclusions: Our risk formula shows potential prognostic value in multiple cancers and manifest pivotal alterations in immune infiltration and biological pathway in pancreatic cancer.

## INTRODUCTION

Pancreatic adenocarcinoma (PAAD) is a serious kind of cancer with high mortality and increasing morbidity worldwide [1]. Likewise, the incidence and mortality rate of PAAD remains on the rise in China but relatively stable in Japan and South Korea [2]. It is not easy to detect PAAD at an early stage until late in progression, thus losing effective treatment opportunities, which is an important reason for the high mortality in pancreatic cancer. Although carbohydrate antigen 19-9 (CA19-9) and carcinoembryonic antigen (CEA) are commonly used in clinical practice for tumor markers, they have limited specificity and sensitivity to screen patients with early pancreatic ductal adenocarcinoma (PDAC) [3]. Therefore, it is essential to identify effective prognostic biomarkers and to establish a valid prognostic model in PAAD.

Exosomes are nanosized (30-150nm), physiologically released extracellular vesicles of endosomal origin, and carry substances such as nucleic acids, proteins, and lipids [4, 5]. Cancer derived exosomes are involved in multiple biological processes, including epithelial-to mesenchymal transition, cell proliferation, and angiogenesis [6–9]. Furthermore, there is a considerable amount of literature on deregulation of lipid metabolism in cancer cells. Considering the increasing evidence that lipid metabolism disorder plays a key role in PAAD patients, it is necessary to comprehensively evaluate the prognostic significance of exosome and lipid metabolism-related genes, which might provide potential prognostic biomarkers. However, studies on this problem have received scant attention in research literature.

This study acquired the transcriptome profiling data of PAAD with clinical information from the TCGA database and GEO. A total of 52 exosome and lipid metabolism-related genes (EALMRGs) were obtained from the GeneCards and MSigDB databases after merging and deduplication. We constructed a prognostic model and implemented external and internal validation to evaluate the effectiveness and availability of the model. Finally, 5 significant prognoses related EALMRGs were identified and verified in GEO. To further explore the potential mechanism and relationship of these genes, Gene Ontology (GO), Kyoto Encyclopedia of Genes and Genomes (KEGG), and protein-protein interaction (PPI) analysis were conducted. We also performed Gene Set Enrichment Analysis (GSEA) and Gene Set Variation Analysis (GSVA) to understand the functional enrichment and differences between high and low-risk groups based on the prognostic model. We constructed a potential prognostic model and identified 5 significant prognosis EALMRGs and explored the potential mechanism. In this way, our study reveals the value of EALMRGs in predicting the prognosis of PAAD patients and provides clues for the specific mechanisms associated with lipid metabolism and cancer.

## MATERIALS AND METHODS

### Data collection and pre-processing

We downloaded high-throughput sequencing RNA data and clinical information on PAAD from the TCGA database (https://portal.gdc.cancer.gov/). Excluding 4 samples of para-carcinoma tissues, a total of 179 PAAD patients were enrolled. RNA sequencing data were transformed from fragments per kilobase per million (FPKM) formats to transcripts per million (TPM) reads for this study. Corresponding clinical information was downloaded from the UCSC Xena browser (http://genome.ucsc.edu) [10]. The count sequencing and clinical data in the TCGA-PAAD dataset were standardized using R packet limma [11]. In addition, two raw gene expression datasets; GSE62452 [12] and GSE57495 [13] downloaded from the GEO database [14] were used to validate further analysis using GEOquery.

Human genes with comprehensive information were provided by the GeneCards database [15]. Based on the term “Exosome” as a keyword and “Protein Coding”, “Relevance score>2” as a screening criterion, we obtained 589 exosome-related genes (ERGs) from the GeneCards database. Furthermore, we also collected 77 ERGs on the Molecular Signatures Database (MSigDB database) [16] website using “Exosome” as the keyword. A total of 628 ERGs were obtained after merging and removing duplicates. Likewise, we used “lipid metabolism” as the keyword on the GeneCards database, and 680 lipid metabolism-related genes (LMRGs) were collected. Meanwhile, 102 LMRGs were obtained on the MSigDB database using keywords “lipid metabolism”. A total of 747 LMRGs were collected after merging and removing duplicates. Finally, we obtained 52 EALMRGs by intersecting 628 ERGs and 747 LMRGs (Supplementary Table 1).

### Construction and evaluation of EALMRGs prognostic models

We selected TCGA-PAAD tumor samples (Table 1) and performed univariate COX regression analysis on overall survival (OS) related EALMRGs by survival packet. A total of 10 prognosis-related EALMRGs were acquired with P<0.1. Based on these 10 EALMRGs, the software package “glmnet” [17] was used to perform the Least absolute shrinkage and selection operator (LASSO) [18] logistic regression analysis with tenfold cross-validation. LASSO regression is a common machine learning technique for constructing prognostic diagnostic models. It is used to solve the problem of overfitting by adding a penalty term (lambda × absolute value of slope), using regularization based on linear regression, and additionally, improve the model’s generalization ability.


$$\begin{array}{l} riskScore=\sum_{i}Coefficient(Hub\;gene_{i})*\\ \;\;\;\;\;\;\;\;\;\;\;\;\;\;\;\;\;\;\;\;\;mRNA\;Expression(Hub\;gene_{i}) \end{array}$$

**Table 1:** Baseline characteristics in TCGA-PAAD.

**Characteristics**	**Overall**
*Pathologic T stage, n (%)*	
T1	7 (4)
T2	24 (13.6)
T3	143 (80.8)
T4	3 (1.7)
*Pathologic N stage, n (%)*	
N0	50 (28.7)
N1	124 (71.3)
*Pathologic M stage, n (%)*	
M0	80 (94.1)
M1	5 (5.9)
*Gender, n (%)*	
Female	80 (44.7)
Male	99 (55.3)
*Age, median (IQR)*	65 (57, 73)
*OS event, n (%)*	
Alive	86 (48)
Dead	93 (52)
*DSS event, n (%)*	
Yes	73 (42.2)
No	100 (57.8)
*PFI event, n (%)*	
Yes	105 (58.7)
No	74 (41.3)

Subsequently, prognostic-related genes were identified as key genes. We drew KM curves and AUC to evaluate the prognosis value of these key genes. A risk factor graph was drawn to show the groups according to each sample’s risk score and survival outcomes in this prognostic model. We also used calibration analysis to evaluate the performance of the prognostic model based on EALMRGs using the rms R package. Decision curve analysis (DCA) is a simple method for evaluating clinical predictive models, diagnostic trials, and molecular markers. We also drew DCA maps to evaluate the predictive effect of the model on the 1-, 3-, and 5-year survival state of PAAD patients using the ggDCA R package.

### Identification of DEGs based on EALMRGs prognostic models

For data analysis, the dataset TCGA-PAAD was corrected and standardized by limma R packet. We calculated the risk score of the TCGA-PAAD sample based on the prognostic model and then divided the samples into high and low-risk groups according to the risk score. Differentially expressed genes (DEGs) between two groups in the TCGA-PAAD dataset were obtained and presented by plotting volcanic maps using ggplot2.package. By selecting DEGs with |logFC| >0 and P.adj<0.05, we drew heat maps and group comparison maps to display the key gene expression in the TCGA-PAAD dataset using pheatmap R packet. In addition, we standardized GSE62452 and GSE57495 from the GEO dataset and selected samples of pancreatic cancer patients to batch merge them into a Dataset-PAAD using the sva package. According to the risk score, we divided Dataset-PAAD into high and low-risk groups and drew a group comparison map. Genes with consistent trends of TCGA-PAAD and Dataset-PAAD were selected as hub genes for subsequent analysis.

### Bulk data download and pre-processing

The clinical phenotype data of PAAD was downloaded from the TCGA database. The samples lacking survival time and survival state were removed, and all those with survival time greater than 0 days were saved. Meanwhile, TCGA expression profile data (log2 (TPM)) was downloaded. Finally, 176 tumor samples were obtained.

The clinical phenotype data of three external validation datasets: PACA_AU, PACA_CA, and PAEN-AU were downloaded from the ICGC database. The samples lacking survival time and survival state were removed, and all those with survival time greater than 0 days were saved. Meanwhile, the corresponding expression profile data (log2 (TPM)) was downloaded.

GDC Pan Cancer (PANCAN) 33 pan-cancer sequencing data and corresponding survival information were downloaded from UCSC Xena to analyze the pan-cancer section.

### scRNA data download

Single-cell sequencing data in the GEO database, GSE155698, was downloaded. It contained 16 pancreatic cancer tissue samples and 3 adjacent normal pancreas samples. The procedure of single-cell sequencing is exerted using the Seurat package in R software.

### Functional similarity analysis

In this study, GO and KEGG enrichment was performed by the clusterProfiler [19–22] R package with a screening criterion of P.adj < 0.1 and a P-value correction method of Benjamin Hochberg (BH). Gene Set Enrichment Analysis (GSEA) and GSVA [23–25] were performed to determine differentially enriched signaling pathways between high and low-risk groups. A function or pathway term with adjusted P.adj < 0.05, and false discovery rate (FDR) < 0.25 was considered statistically significant. In our study, the PPI network was constructed using the STRING database [26] to obtain the interactions among hub genes in EALMRGs, and the least required interaction score was 0.4. Furthermore, we used the GeneMANIA website [27] to predict the functionally similar genes of the screened hub genes and constructed an interaction network. We used the miRDB database [28] to predict miRNAs that interacted with Hub genes and then plotted the mRNA-miRNA interaction network using data with Target Score>90. Besides, we searched for TF that binds to hub genes through the CHIPBase and hTFtarget database, and the common parts were retained in both databases [29, 30].

### Cell culture

Pancreatic carcinoma cell lines of Homo sapiens, including PANC-1, SW1990, BXPC-3, CFPAC1, ASPC1, and normal pancreatic cell line HPDE6-C7 were obtained as a gift from Dr. Deyu Zhang in Changhai Hospital. Cells were cultured in DMEM with 10% fetal bovine serum (Gibco, USA), 100 μg/mL streptomycin, and 100 U/mL penicillin (Invitrogen, USA) at 37° C under 5% CO2 and 1% O^2^. All the following experiments were independently repeated three times.

### RNA extraction and qRT-PCR of the hub genes

Trizol reagent (Invitrogen, Carlsbad, CA, U.S.A.) was used to extract total RNA from the cells according to the manufacturer’s protocol. Superscript II reverse transcriptase and random primers were used to synthesize cDNA. Quantitative real-time PCR (qRT-PCR) was conducted on the ABI 7900HT Sequence Detection System with SYBR-Green dye (Applied Biosystems, Foster City, CA, U.S.A.). All primers were as following: GAPDH Forward: 5′-GGACCTGACCTGCCGTCTAG-3′, Reverse: 5′-GTAGCCCAGGATGCCCTTGA-3′, ABCB1: Forward: 5′-CCCATCATTGCAATAGCAGG-3′and Reverse: 5′-GTTCAAACTTCTGCTCCTGA-3′, CAP1: Forward: 5′-GAAGGTGAAGGT CGGAGTC-3′and Reverse: 5′-CCCGAATCACATTCTCCAAGA A-3′, EGFR: Forward: 5′-GGACGACGTGGTGGATGC-3′and Reverse: 5′-GGCGCCTGTGGGGTCTGAGC-3′, PPARG: Forward: 5′- ACCAAAGTGCAATCAAAGTGGA-3′and Reverse: 5′- ATGAGGGAGTTGGAAGGCTCT-3′, SNCA: Forward: 5′- AAGAGGGTGTTCTCTATGTAGGC-3′ and Reverse: 5′- GCTCCTCCAACATTTGTCACTT-3′. The reaction parameters included a denaturation program (10 min at 95° C), followed by an amplification and quantification program over 45 cycles (15 s at 95° C and 34 s at 60° C). Each sample was tested in triplicates, and each sample underwent a melting curve analysis to check for the specificity of amplification. The expression level was determined as a ratio between the hub genes and the internal control GAPDH in the same mRNA sample and calculated by the comparative CT method. Expression levels of hub genes were calculated by the 2^−δδ^Ct method.

### Statistical analysis

All data analyses in this study were performed and visualized in R software (Version 4.1.2) or GraphPad Prism (Version 8). The Wilcoxon rank sum test was used to compare the two groups of continuous variables, and the statistical significance of normal distribution variables was estimated through an independent Student t-test. LASSO regression analysis was based on the R package glmnet and ROC [31] using the R package pROC. If not particularly specified, the results were calculated using Spearman correlation analysis to calculate the correlation coefficients between different molecules. All P-statistics were bilateral, with a P-value<0.05 as a statistically significant criterion.

## RESULTS

### Merge and correction of dataset in GEO

**Figure 1:**
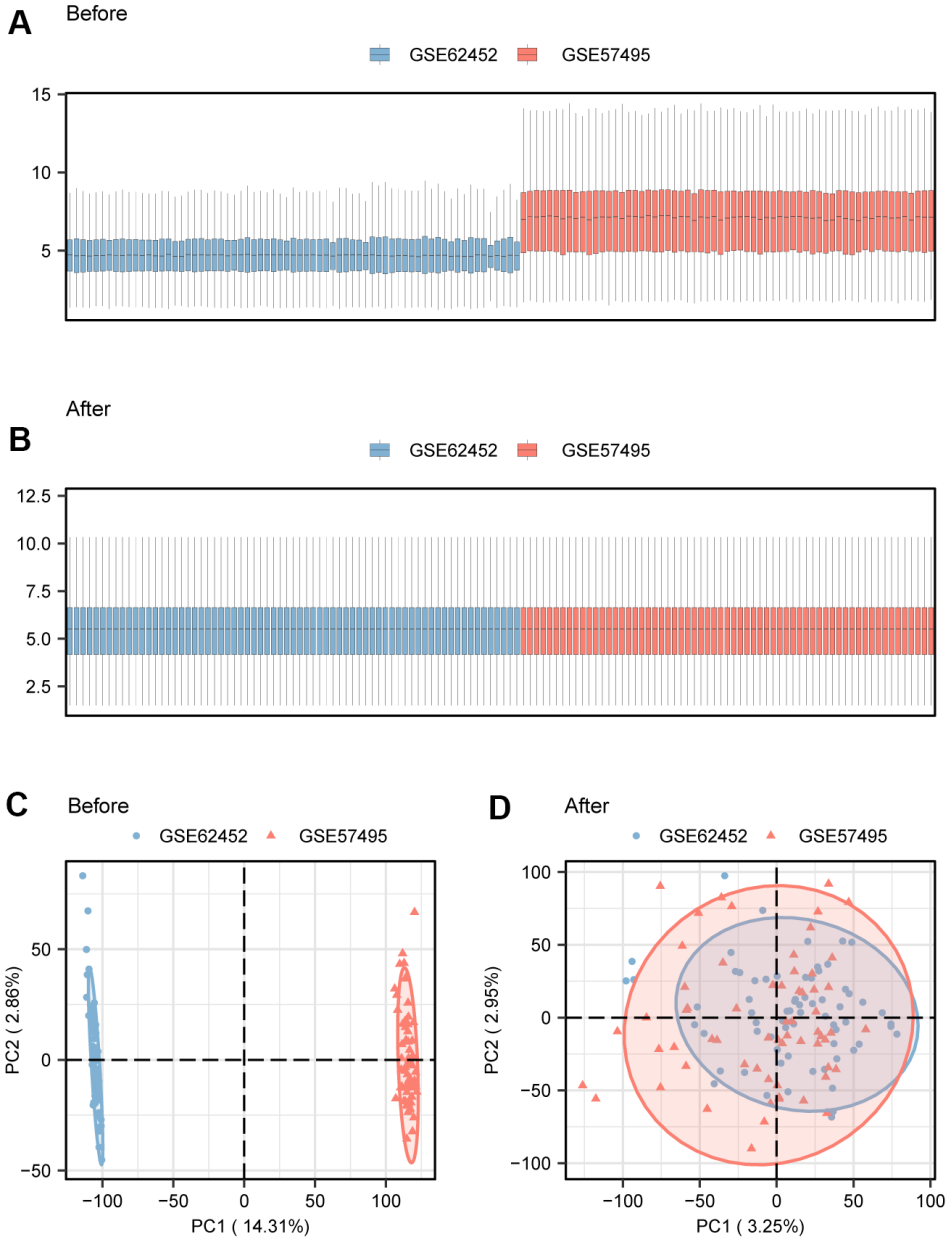
**Merge and correction of dataset.** (**A**) Boxplot to show merged dataset before batch processing. (**B**) Boxplot to show merged dataset after batch processing. (**C**) PCA to show merged dataset before batch processing. (**D**) PCA to show merged dataset after batch processing. PCA, Principal Component Analysis.

The gene expression matrix of GSE62452 and GSE57495 datasets from GEO was firstly background corrected and data normalized. We obtained Dataset-PAAD using the sva R package to remove batch effects of the combined two datasets. We also compared the datasets before and after the removal of batch effects through distribution Boxplot (Figure 1A, 1B) and Principal Component Analysis (PCA) (Figure 1C, 1D). The results indicated that the batch effect of Dataset-PAAD samples was eliminated after processing.

### Construction of EALMRGs prognostic models

**Figure 2:**
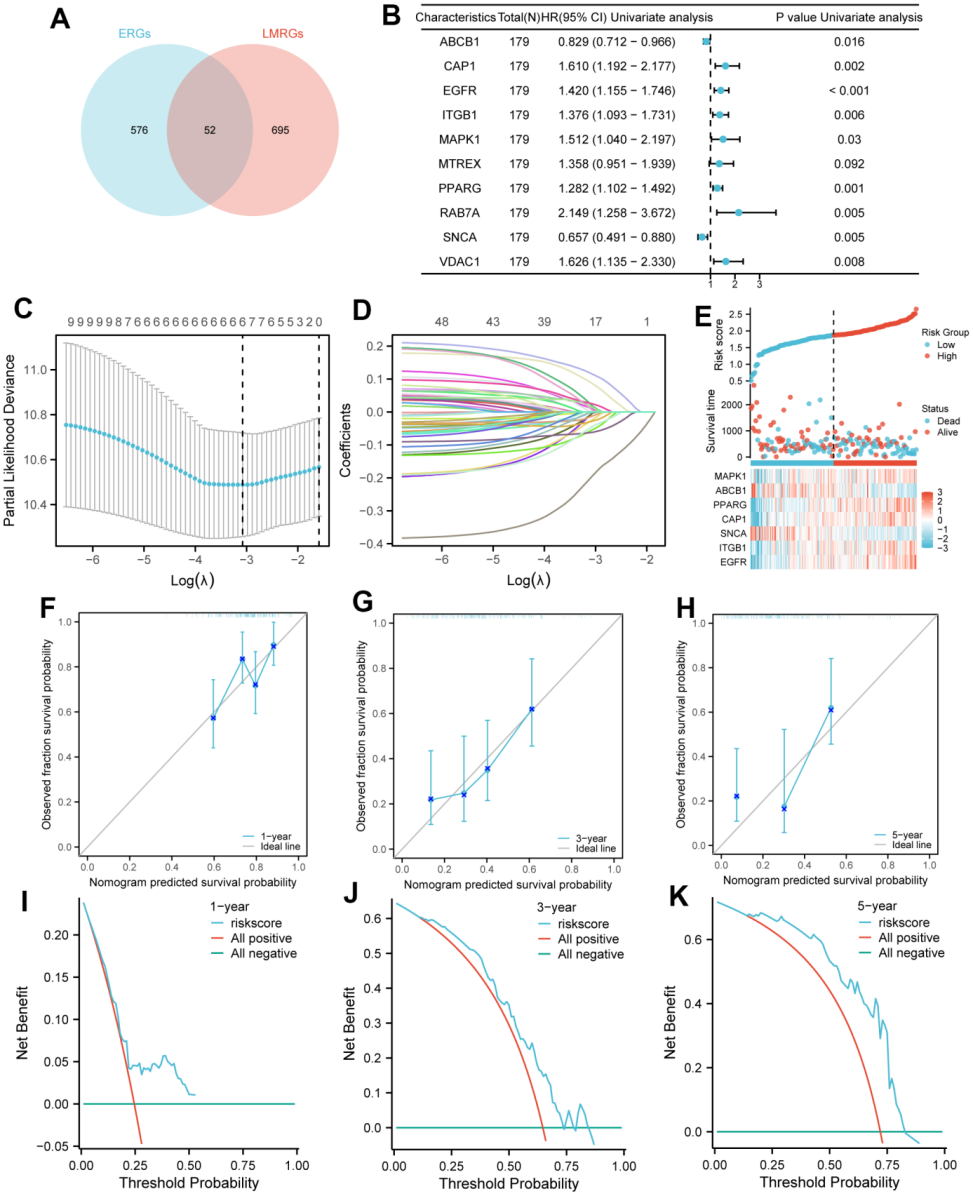
**Model establishment for prognosis.** (**A**) Venn diagram: the blue circle on the left includes the 576 ERGs, the red circle on the right includes the 695 LMRGs, and the intersection of the two circles includes the 52 EALMRGs. (**B**) Forest plot to show the result of univariate Cox regression analysis. (**C**) The confidence interval under each lambda. (**D**) The changing trajectory of each independent variable. (**E**) Risk score nomogram consists of 3 parts, including risk group, survival outcomes, and heatmap. (**F**–**H**) The calibration curves of the nomogram are at 1-year (**F**), 3-year (**G**), and 5-year (**H**), respectively. The X-axis in curves represented nomogram predicted survival probability and Y-axis represented observed fraction survival probability. (**I**–**K**) DCA diagrams of the models for 1-year (**I**), 3-year (**J**), and 5-year (**K**), respectively. The X-axis in DCA diagrams represents Threshold Probability and the Y-axis represents Net Benefit. LASSO, Least absolute shrinkage and selection operator; TCGA, The cancer genome atlas; OS, Overall survival; DCA, decision curve analysis; LASSO, least absolute shrinkage and selection operator.

We obtained 52 EALMRGs by intersecting 628 ERGs and 747 LMRGs (Figure 2A). A total of 10 prognosis-related EALMRGs were obtained using univariate Cox regression analysis with P < 0.1 in EALMRGs from TCGA-PAAD, including *ABCB1*, *CAP1*, *EGFR*, *ITGB1*, *MAPK1*, *MTREX*, *PPARG*, *RAB7A*, *SNCA*, *VDAC1* (Table 2), and forest plot was drawn to show the results (Figure 2B). We used LASSO regression analysis to construct a LASSO prognostic model based on the expression of these 10 genes. The optimal lambda value for the evaluation index corresponds to 7 genes with non-zero coefficients (Figure 2C), and a LASSO prognostic variable trajectory map was drawn (Figure 2D). These 7 genes were identified as key genes, including *ABCB1*, *CAP1*, *EGFR*, *ITGB1*, *MAPK1*, *PPARG*, and *SNCA*. We also visualized the high and low expression in the LASSO prognostic model with risk score, consisting of 3 parts, including risk group, survival outcomes, and heatmap (Figure 2E).

**Table 2:** Univariate and multivariate Cox regression.

		**Univariate analysis**		**Multivariate analysis**	
**Characteristics**	**Total (N)**	**HR (95% CI)**	**P-value**	**HR (95% CI)**	**P-value**
*ABCB1*	179	0.829 (0.712 - 0.966)	0.016	0.783 (0.632 - 0.970)	0.025
*CAP1*	179	1.610 (1.192 - 2.177)	0.002	0.932 (0.543 - 1.602)	0.800
*EGFR*	179	1.420 (1.155 - 1.746)	< 0.001	1.161 (0.859 - 1.568)	0.331
*ITGB1*	179	1.376 (1.093 - 1.731)	0.006	1.054 (0.655 - 1.697)	0.829
*MAPK1*	179	1.512 (1.040 - 2.197)	0.030	1.925 (0.924 - 4.008)	0.080
*MTREX*	179	1.358 (0.951 - 1.939)	0.092	0.998 (0.581 - 1.716)	0.995
*PPARG*	179	1.282 (1.102 - 1.492)	0.001	1.052 (0.864 - 1.281)	0.614
*RAB7A*	179	2.149 (1.258 - 3.672)	0.005	0.984 (0.428 - 2.260)	0.969
*SNCA*	179	0.657 (0.491 - 0.880)	0.005	0.695 (0.489 - 0.990)	0.044

### Evaluation of prognostic EALMRGs genes

In our study, we conducted a 1-, 3- and 5-year prognostic calibration analysis based on the LASSO model and plotted a calibration curve. The results revealed that the 1- and 3-year prediction effect was better than that of the 5-year (Figure 2F–2H). Meanwhile, we used DCA diagrams to evaluate the clinical utility of the LASSO prognostic model at 1-, 3- and 5-year, and the results presented that the predictive value of the model at 5-year was better than that at 1- and 3-year (Figure 2I–2K).

**Figure 3:**
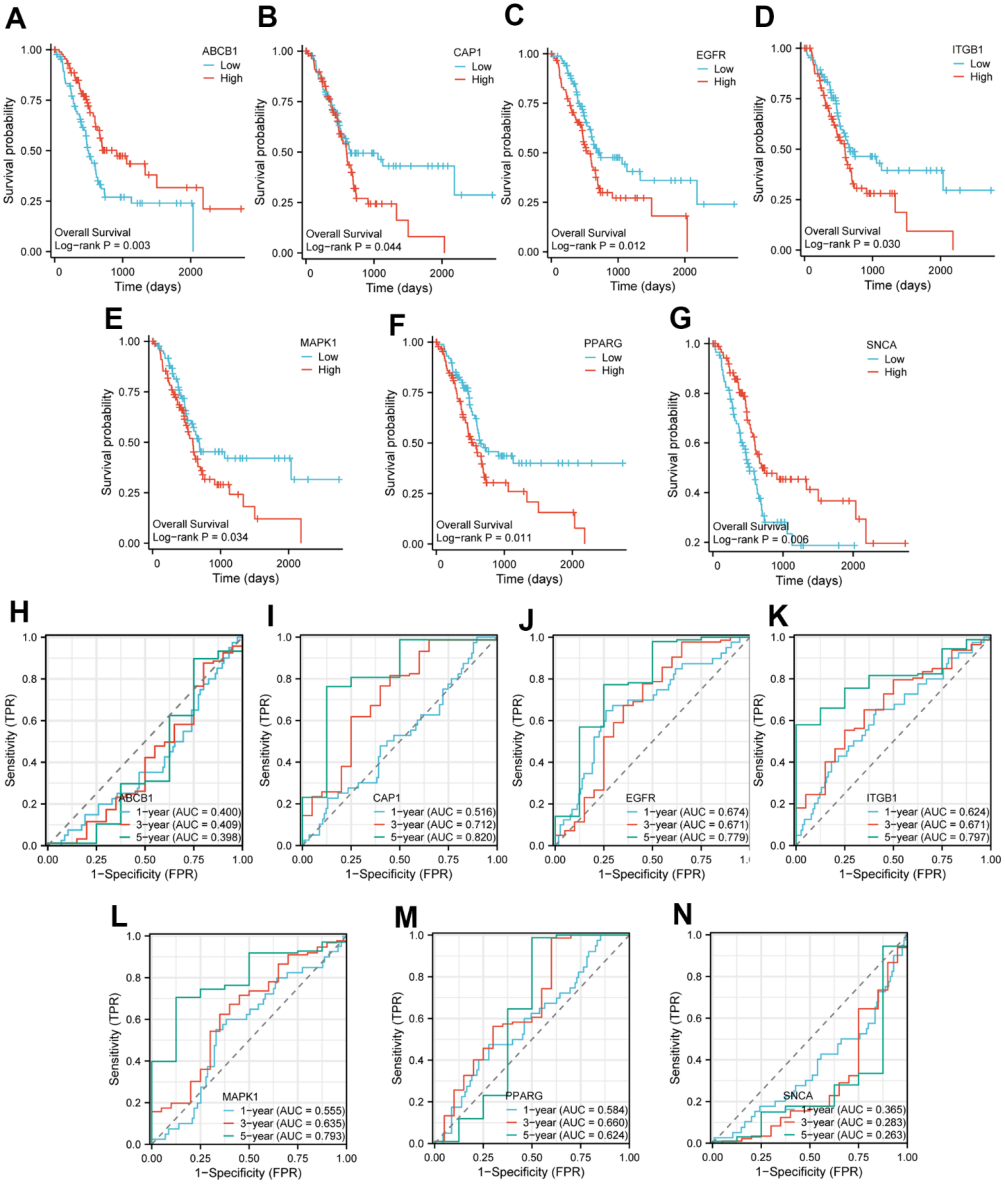
**KM curve and time ROC of key genes in TCGA-PAAD.** (**A**–**G**) The KM curve of the high and low expression groups of Key genes in TCGA-PAAD. Patients with high expression of *ABCB1* (**A**) and *SNCA* (**G**) had significantly longer overall survival; patients with high expression of *CAP1* (**B**), *EGFR* (**C**), *ITGB1* (**D**), *MAPK1* (**E**) and *PPARG* (**F**) had significantly shorter overall survival. (**H**–**N**) The time ROC curve of the high and low expression groups of Key genes in TCGA-PAAD. ROC curve showed the efficiency of 7 key gene expression levels to predict the prognosis over time. The X-axis represents a false positive rate, and the Y-axis represents a true positive rate. OS, Overall survival; KM, Kaplan–Meier; ROC, receiver operating characteristic curve.

**Figure 4:**
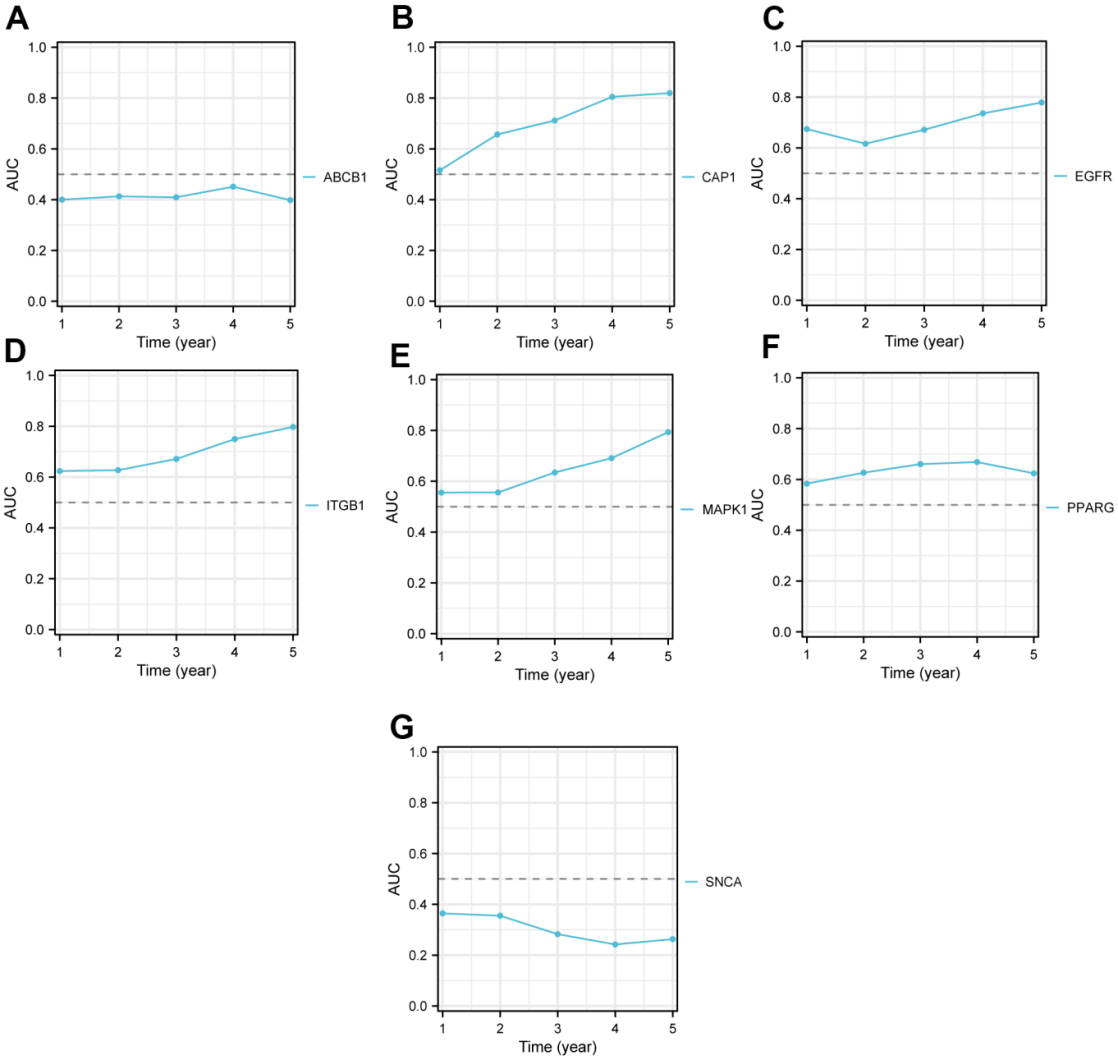
**The time AUC curve of the high and low expression groups of Key genes in TCGA-PAAD.** The AUC of *ABCB1* (**A**) is less than 0.5 over time, which indicates they were protective factors, and the closer the AUC value is to 0, the better the prediction performance. The AUC of *CAP1* (**B**), *EGFR* (**C**), *ITGB1* (**D**), *MAPK1* (**E**), and *PPARG* (**F**) are all over than 0.5 over time, which indicates they were risk factors, and the closer the AUC value is to 1, the better the prediction performance. The AUC of *SNCA* (**G**) is also less than 0.5 over time. The X-axis represents time, and Y-axis represents the AUC value. TCGA, The cancer genome atlas; AUC, Area Under Curve.

Furthermore, according to these 7 key genes (*ABCB1*, *CAP1*, *EGFR*, *ITGB1*, *MAPK1*, *PPARG*, *SNCA*), we plotted the KM curves to show the survival state of high and low expression groups in tumor samples from the TCGA-PAAD dataset. The result of KM curves suggested that the survival rate of PAAD patients in the high-expression group of genes *ABCB1* and *SNCA* was higher than that in the low-expression group over time, while the survival rate of PAAD patients in the high-expression group of *CAP1*, *EGFR*, *ITGB1*, *MAPK1*, *PPARG* was lower than that in the low-expression group over time (Figure 3A–3G). We also plotted time ROC curves of high and low gene expression groups (Figure 3H–3N) and the trend of AUC of 7 key genes and prognostic models over time (Figure 4A–4G). All 7 genes performed well in predicting the prognosis of PAAD patients at 5-year, with EGFR and ITGB1 having better overall predictive effects than other genes with certain accuracy in LASSO models.

### Validation of risk models using external data sets

**Figure 5:**
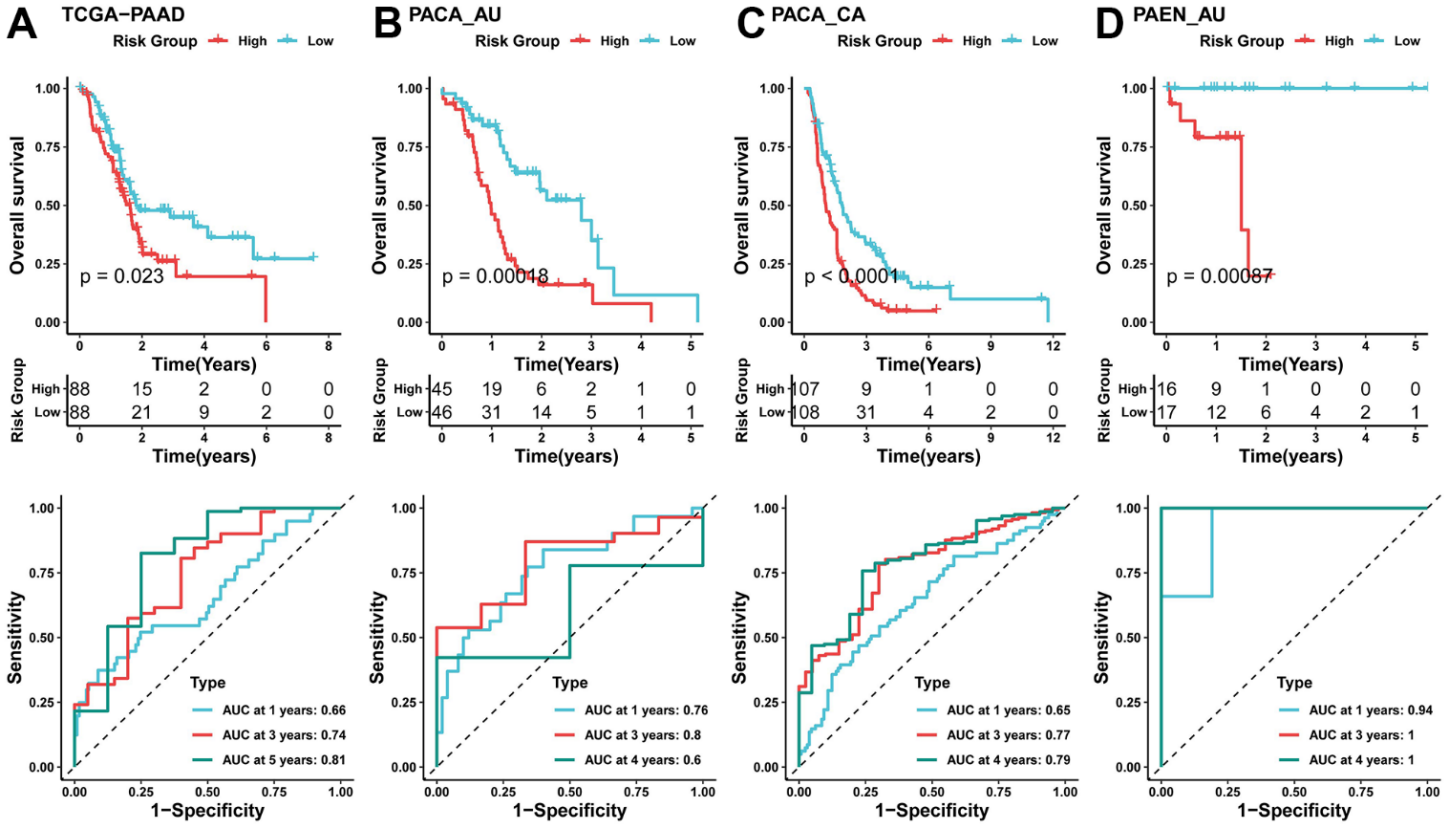
**Validation of risk models using external data sets.** (**A**) KM curve (top) of TCGA-PAAD dataset, Time-dependent area under the receiver operating characteristic curve (AUC) at 1-, 3-, and 5-year in the A TCGA-PAAD (bottom); (**B**–**D**) KM curve (top), Time-dependent area under the receiver operating characteristic curve (AUC) at 1-, 3-, and 4-year in the B PACA_AU, C PACA_CA, D PAEN_AU (bottom).

We used these 5 key genes to construct a risk model on the TCGA-PAAD dataset, calculated the risk score for each patient, and divided into high and low-risk groups by the median. According to the KM curve, it could be seen that the survival of patients in the high-risk group was poor, and the risk model had good value in predicting the prognosis survival time (Figure 5A). To verify the generalization of the model, we evaluated the model on three external independent datasets. Through the prediction of the model, there were significant differences in survival between high and low-risk groups, and it has good predictive performance for patient prognosis and survival time (Figure 5B–5D). Moreover, the mRNA level of ABCB1 and SNCA were identified with significant downregulation in pancreatic cancer cell lines compared to normal pancreatic cells. Additionally, the mRNA level of CAP1, EGFR and PPARG were identified with significant downregulation in pancreatic cancer cell lines compared to normal pancreatic cells (Supplementary Figure 1A).

### Immune microenvironment analysis in high and low-risk groups

**Figure 6:**
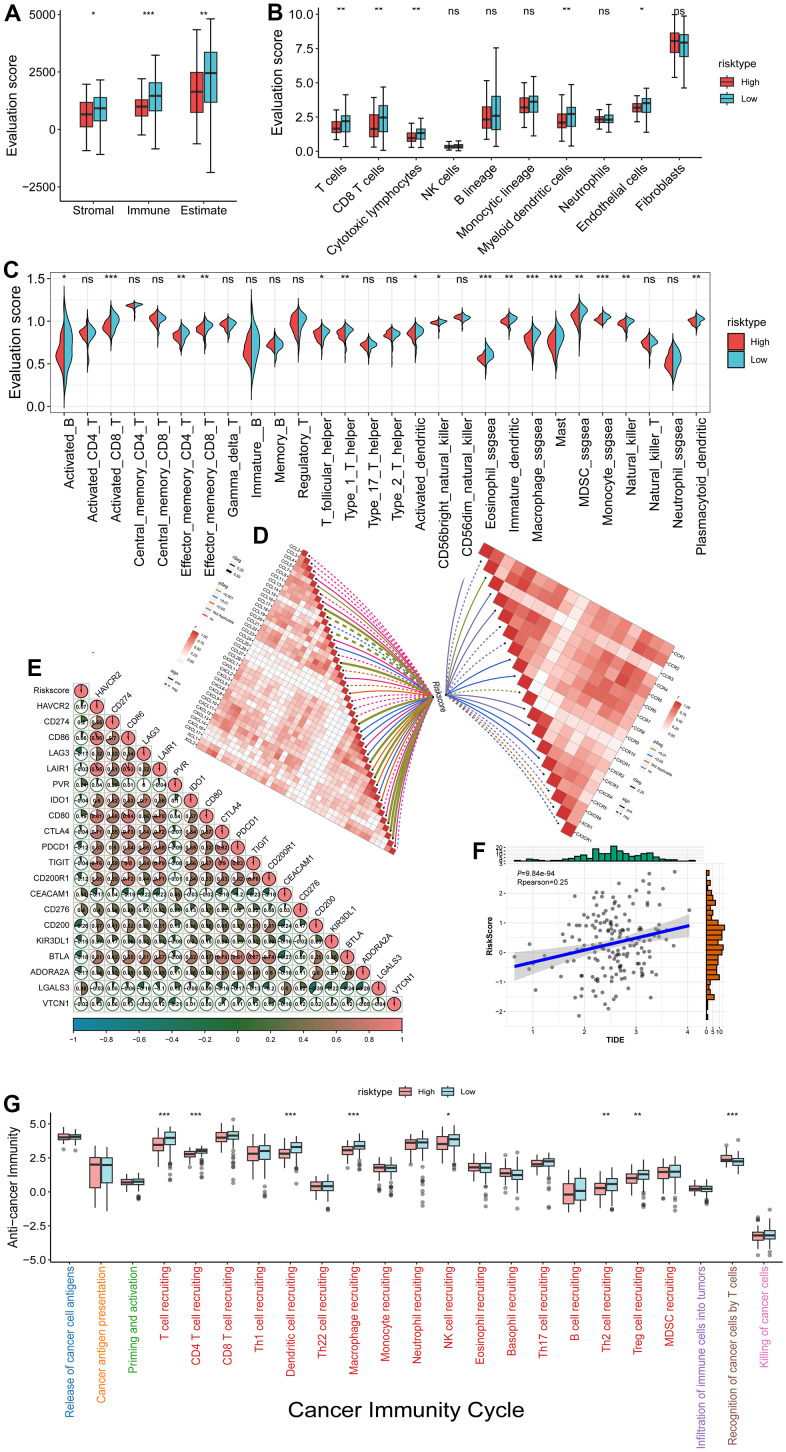
**Immune microenvironment analysis in high and low risk groups.** (**A**) Comparison of the Estimate score in high and low-risk groups; (**B**) MCP-counter immune cell infiltration score was compared between high and low-risk groups; (**C**) The ssgsea calculated 28 kinds of immune cell infiltration scores in high and low-risk groups. (**D**) Correlation analysis between risk score and chemokines and receptors; (**E**) Correlation analysis between risk score and 20 kinds of immune checkpoints; (**F**) Correlation analysis between risk score and TIDE score (immune escape); (**G**) TIP calculated comparison of tumor immune cycle step scores in high-low risk groups. *<0.05 **<0.01 ***<0.001.

To further analyze the differences in the immune microenvironment in patients with high and low-risk, we compared the immune scores predicted by ESTIMATE. We found lower levels of immune infiltration in high-risk patients (Figure 6A). We then used the MCP-counter algorithm to examine the approximate infiltration of cell types, and the results showed that multiple immune cell types had less infiltration in high-risk patients (Figure 6B). The ssgsea algorithm was then used to analyze the relationship between the high-low risk group and 28 kinds of immune cell scores, and we found that the low-risk group had higher immune cell scores (Figure 6C). The infiltration of immune cells is directly related to patient survival, and some studies have shown a certain correlation between chemokines and immune cell infiltration. Therefore, we analyzed the correlation between risk scores and chemokines and their receptors (Figure 6D).

The function of immune cells is affected by immune checkpoints. We then analyzed the correlation between risk score and immune checkpoints (Figure 6E) and found a positive correlation with most immune checkpoints.

Subsequently, we analyzed the potential clinical effects of immunotherapy evaluated by using TIDE (http://tide.dfci.harvard.edu/) software in our defined high-risk groups. The higher the TIDE prediction score, the higher the likelihood of immune escape, indicating a lower likelihood of patients benefiting from immunotherapy. It was found that the higher the risk score, the higher the TIDE score (Figure 6F).

More profoundly, to examine the differences in immune stages, we used the TIP analysis tumor immune phenotype (TIP) database (http://biocc.hrbmu.edu.cn/TIP), a web-based tool that can evaluate the immune microenvironment based on the cancer immune cycle. We calculated the scores for each of the seven steps of the immune cycle in each sample (one step for each color from left to right) and found a higher immune step score (Figure 6G) in the low-risk group.

### Analysis of prognostic value of risk model in pan-cancer

**Figure 7:**
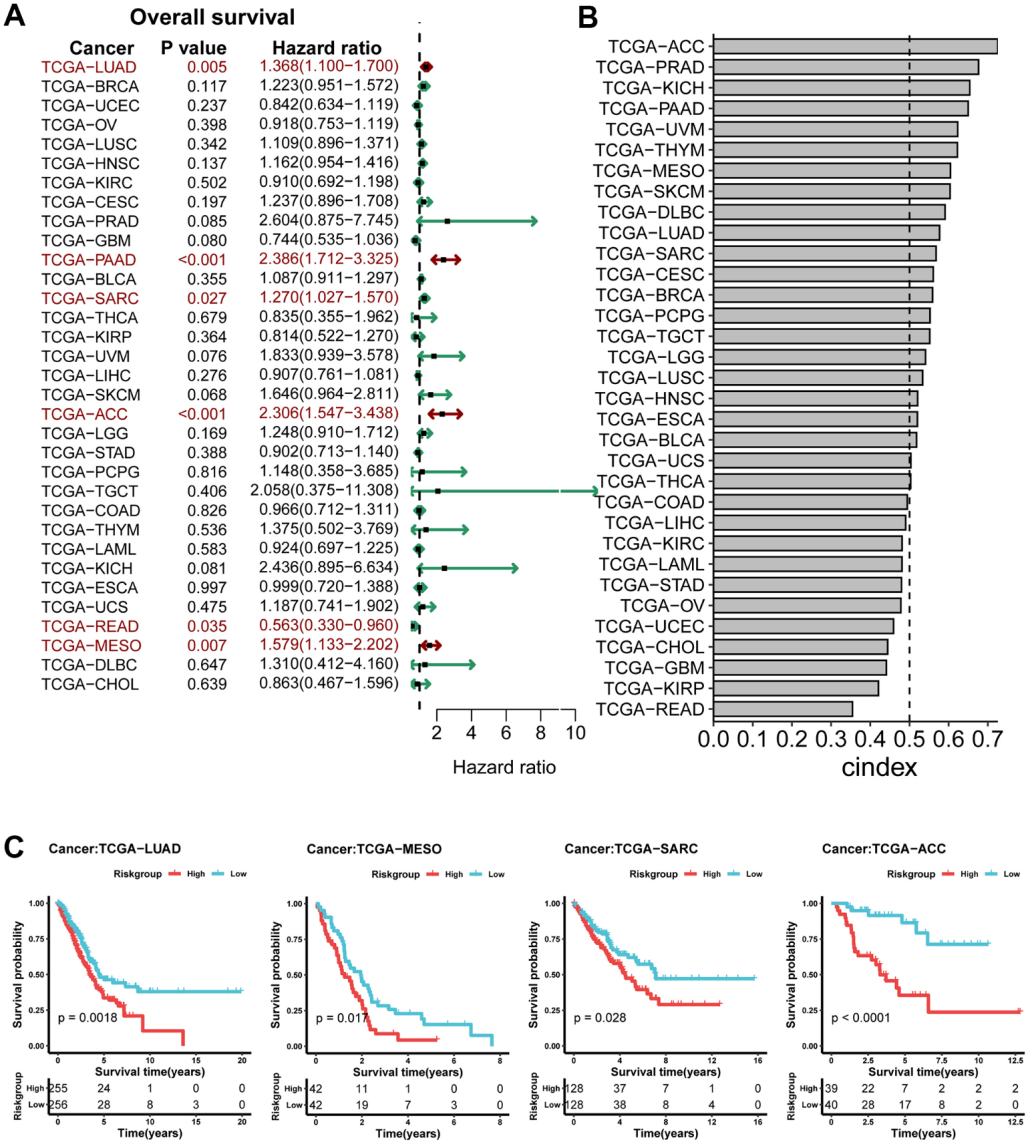
**Analysis of prognostic value of risk model in pan-cancer.** (**A**) Analysis of the relationship between survival model and OS in pan-cancer; (**B**) Cindex of survival model on pan-cancer; (**C**) KM curves of TCGA-LUAD, TCGA-MESO, TCGA-SARC, TCGA-ACC.

We evaluated the model on 33 pan-cancer data, and through univariate cox analysis, we found that it was significant in 5 datasets other than PAAD, and as a risk prognostic factor in 4 datasets (Figure 7A). Simultaneously, a favorable C-index (Figure 7B) was shown in multiple datasets. Subsequently, K-M plot was plotted for these four datasets, and patients in the high-risk group also have poorer prognoses in multiple cancers (Figure 7C). Moreover, we used the GSVA package to perform a pathway enrichment score on the pan-cancer dataset for the pathways in KEGG. Subsequently, correlation analysis was conducted based on their respective risk scores to screen for pathways (P-value < 0.05), and then pathways that were retained for more than 10 cancer species were screened and shown in Supplementary Figure 1B.

### Discovery of DEGs based on EALMRGs prognostic models

**Figure 8:**
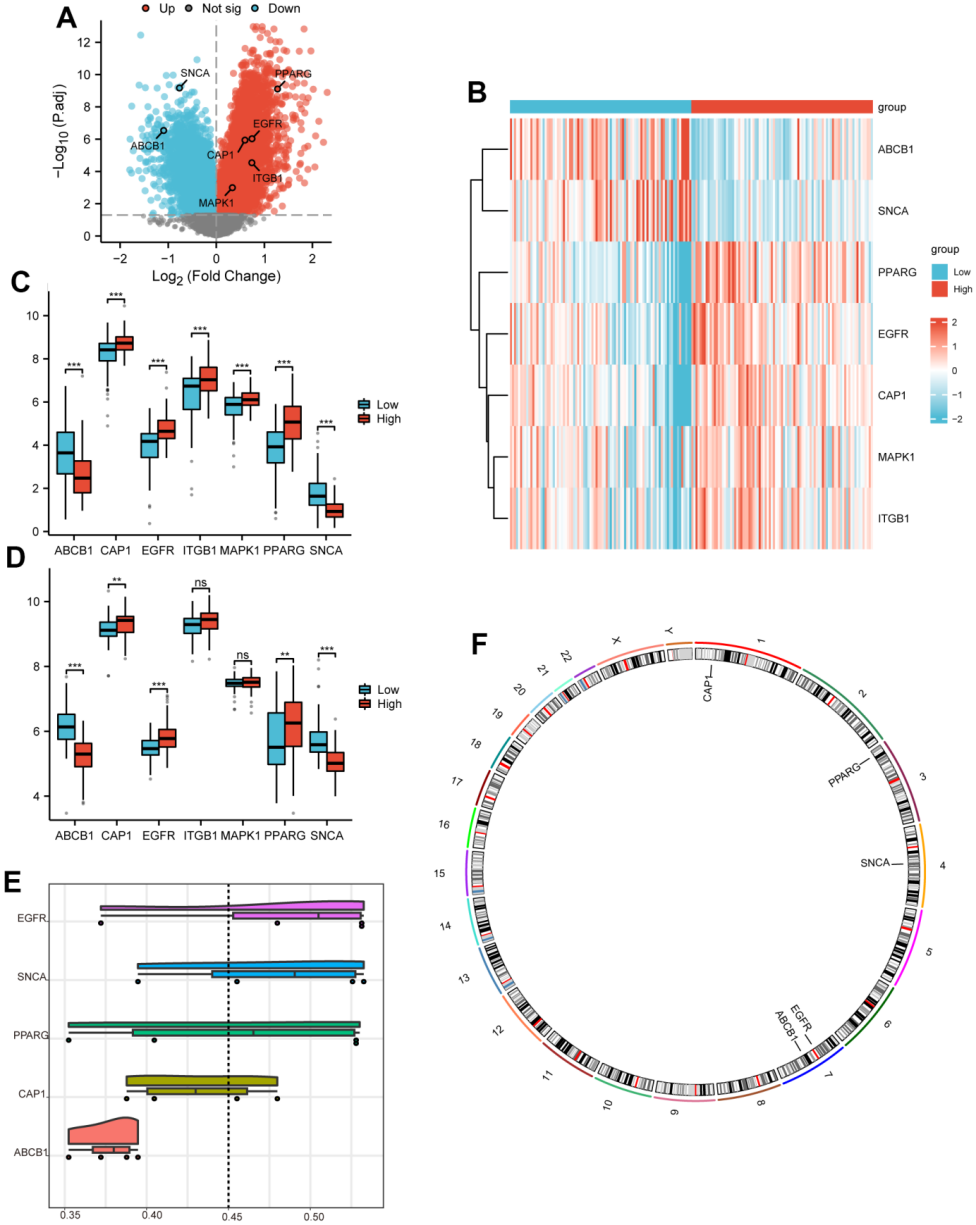
**Differential expression and functional similarity analysis.** (**A**) Volcano plot of DEGs between high and low-risk groups in TCGA-PAAD, mapping 8016 upregulated genes (red dots) and 5254 downregulated genes (blue dots). No significantly changed genes are marked as gray dots. (**B**) The heatmap to show high and low-risk groups and 7 key gene expressions. (**C**, **D**) Boxplots to show the expression difference of *ABCB1*, *CAP1*, *EGFR*, *ITGB1*, *MAPK1*, *PPARG*, and *SNCA* between high and low-risk groups in TCGA-PAAD dataset (**C**) and Dataset PAAD (**D**). (**E**) Raincloud plots to show the functional similarity analysis of 5 hub genes; X-axis represents a similarity score, and the larger the value, the higher the functional similarity with other genes. (**F**) Chromosomal mapping of 5 Hub genes. Ns represents P-value ≥ 0.05, with no statistically significant difference; * represents P-value < 0.05, with a statistically significant difference; ** represents P-value < 0.01; *** represents P-value < 0.001.

We calculated the risk scores of the dataset samples from TCGA-PAAD based on the LASSO regression results. According to the risk scores, samples from TCGA-PAAD and Dataset-PAAD were divided into low and high-risk groups. Subsequently, we performed differential analysis on the processed TCGA-PAAD dataset. A total of 13270 DEGs were identified with |logFC|>0 and P.adj < 0.05 and included 8016 upregulated and 5254 downregulated genes. The DEGs were visualized by the volcano plot (Figure 8A).

We also analyzed the differential expression of 7 key genes (*ABCB1*, *CAP1*, *EGFR*, *ITGB1*, *MAPK1*, *PPARG*, *SNCA*) between low and high-risk groups in the TCGA-PAAD dataset and drew a heatmap using R packet (Figure 8B). The differential expression of key genes can be seen in Table 3. Boxplots were drawn for key genes in the TCGA-PAAD dataset and Dataset-PAAD (Figure 8C, 8D). The expression trends of all key genes in both datasets were consistent between high and low-risk groups.

According to Figure 8D, 5 genes (*ABCB1*, *CAP1*, *EGFR*, *PPARG*, *SNCA*) showed statistical significance in Dataset-PAAD, selected as hub genes for subsequent analysis.

**Table 3:** Key genes DEA in TCGA-PAAD.

**GENE**	**logFC**	**AveExpr**	**t**	**P-value**	**P.adj**	**B**
*SNCA*	-0.76663	1.396627692	-7.459	3.71E-12	6.79E-10	17.23201
*PPARG*	1.272794	4.420288301	7.424397	4.53E-12	7.81E-10	17.03936
*ABCB1*	-1.09405	3.098367518	-6.05659	8.08E-09	2.91E-07	9.838242
*EGFR*	0.741565	4.344547598	5.763118	3.58E-08	9.45E-07	8.413015
*CAP1*	0.595678	8.441607674	5.713291	4.59E-08	1.15E-06	8.175759
*ITGB1*	0.738944	6.717817488	4.87041	2.45E-06	2.90E-05	4.386726
*MAPK1*	0.334392	5.940672572	3.822517	0.000183	0.001014	0.336601

DEA, Differential Expression Analysis; TCGA, The cancer genome atlas.

We calculated semantic similarity among GO terms, sets of GO terms, gene products, and gene clusters using the GOSemSim.R package to analyze the functional similarity of these 5 hub genes. We then visualized the functional similarity analysis results through Raincloud plots (Figure 8E). The results revealed that EGFR had better functional similarity than other hub genes. We also used the RCircos package to annotate the location of 5 hub genes on human chromosomes (Figure 8F). As shown in the graph, these hub genes were mainly distributed on chromosomes 1, 3, 4, and 7, among which chromosome 7 was distributed with 2 hub genes. This indicated that these hub genes had close positions on human chromosomes and were also closely related at the genomic level.

### Pancreatic cancer single cell dimension reduction annotation

Firstly, by setting each gene to be expressed in at least 3 cells and at least 200 genes per cell, the single-cell data was filtered to obtain 55339 cells. Moreover, PercentageFeatureSet function was used to calculate the proportion of mitochondria and rRNA to ensure that the expressed genes of each cell are greater than 200 and less than 10,000, and Supplementary Table 1 shows the count of cells before and after filtration. The Supplementary Figure 2A indicates that UMI is significantly correlated with the amount of mRNA, while UMI/mRNA is not significantly correlated with the content of mitochondrial genes. Supplementary Figure 2B indicates the violin diagram before (TOP) and after (Bottom) quality control.

**Figure 9:**
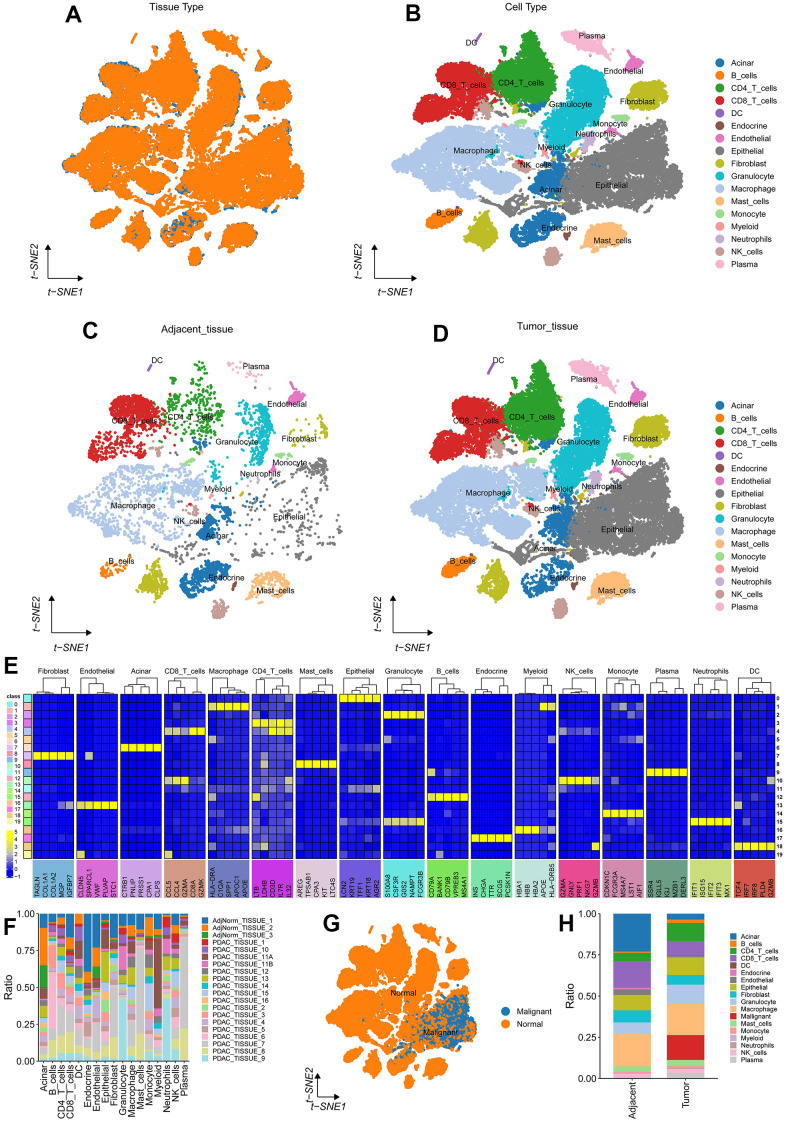
**Pancreatic cancer single cell dimension reduction annotation.** (**A**, **B**) tSNE on Tumor(yellow)/Adjacent(blue) and all patient tissues. (**C**, **D**) tSNE on 3 adjacent/normal pancreas (left) and 16 PDA patient (right) tissues. (**E**) Top 5 marker basis for each cell type; (**F**) Cell count statistics for each sample; (**G**) Copykat prediction results; (**H**) The proportion of various cell types adjacent to the tumor.

Further, we standardized the data for each of the three samples by log-normalization, searched for highly variable genes (based on variance stabilization transformation (“vst”) to identify variable features) by FindVariableFeatures function, scaled all genes by ScaleData function and found anchor points by PCA dimensionality reduction with RunPCA (Supplementary Figure 2C). Dim=35 was selected, and finally, the Find Neighbors and FindClusters functions were used to cluster cells (Resolution=0.3), and a total of 20 subgroups (Supplementary Figure 3) were obtained. At the same time, the RunTSNE function was used to conduct TSNE dimension reduction analysis on 51492 cells. the classical immune cell marker (The markers used were shown in Supplementary Figure 4) reported in the literature was combined with the SigleR algorithm to annotate 20 subgroups of cells, and finally 17 cell types were obtained and visualized according to sample source and cell type (Figure 9A–9D).

Subsequently, we used the FindAllMarkers function to screen marker genes for 17 cell types using logfc=0.35 (multiple of differences) and Minpct=0.35 (smallest expression ratio of differential genes) and performed the screening with a corrected p<0.05. Here, we only show the expression of the top 5 marker genes with the most outstanding contributions in each subpopulation (Figure 9E), and the results of marker genes are shown in the table scRNA_marker_gene.txt. Further, we analyzed the proportion of these 17 types of cells in each sample (Figure 9F).

To further identify malignant cells, we used copykat algorithm to predict malignant cells (Figure 9G, 9H).

### Hub gene was analyzed at the single-cell level

**Figure 10:**
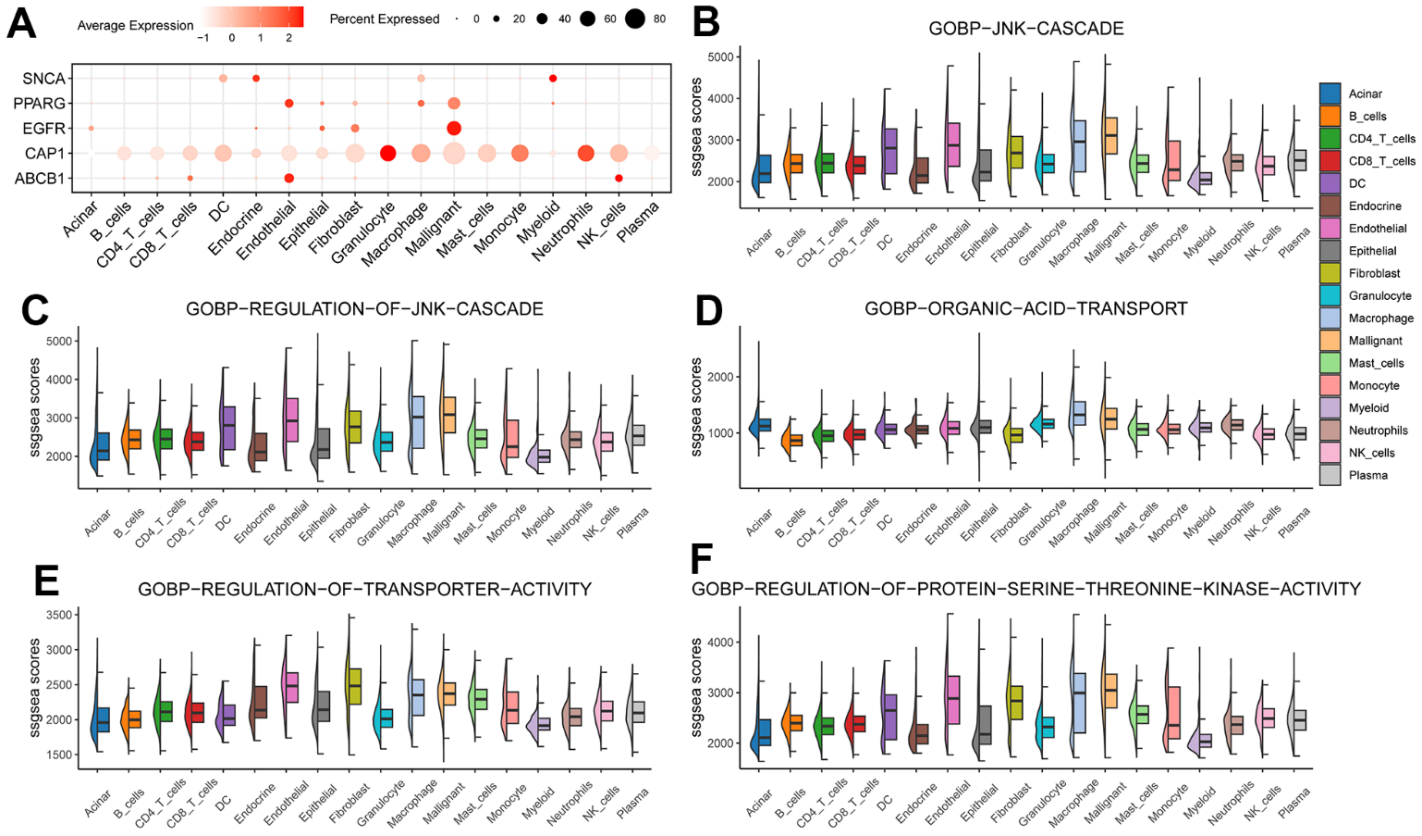
**Hub gene was analyzed at the single-cell level.** (**A**) Hub gene expression in individual cell types; (**B**–**F**) 5 BP enrichment scores in each cell type.

We first examined the expression of hub gene in different cell types (Figure 10A), and then based on the 5 BP results enriched by hub gene on Bulk data, we used the ssgsea algorithm to score these 5 BP in each cell type (Figure 10B–10F).

### Functional enrichment analysis of Hub genes and DEGs

The function of these 5 hub genes was predicted by analyzing GO and KEGG. The GO annotations consisted of three parts, including BP, CC, and MF with criterion P.adj < 0.1, which were used to analyze the functional enrichment of genes. These 5 hub genes were mainly related to BP of regulation of JNK cascade (GO:0046328), JNK cascade (GO:0007254), regulation of transporter activity (GO:0032409), organic acid transport (GO:0015849), regulation of protein serine/threonine kinase activity (GO:0071900) in GO annotation analysis. These genes were also mainly associated with cell cortex (GO:0005938), vesicle lumen (GO:0031983), platelet alpha granule membrane (GO:0031092), focal adhesion (GO:0005925), cell-substrate junction (GO:0030055) in CC and actin binding (GO:0003779), ubiquitin protein ligase binding (GO:0031625), prostaglandin receptor activity (GO:0004955), ubiquitin-like protein ligase binding (GO:0044389), and prostanoid receptor activity (GO:0004954) in MF. The results of GO annotations can be seen in Table 4. KEGG analysis was conducted to explore the relationship between hub genes and signaling pathways. The results indicated that hub genes were not enriched in the KEGG pathway.

**Table 4:** GO enrichment analysis results of hub genes.

**ONTOLOGY**	**ID**	**Description**	**GeneRatio**	**BgRatio**	**P.adj**	**q-value**
**BP**	GO:0046328	regulation of JNK cascade	2023/1/5	141/18800	0.06017	0.01692
	GO:0007254	JNK cascade	2023/1/5	175/18800	0.06518	0.01833
	GO:0032409	regulation of transporter activity	2023/3/5	305/18800	0.00793	0.00223
	GO:0015849	organic acid transport	2023/3/5	318/18800	0.00793	0.00223
	GO:0071900	regulation of protein serine/threonine kinase activity	2023/3/5	372/18800	0.0087	0.00245
	GO:0032768	regulation of monooxygenase activity	2023/2/5	57/18800	0.00916	0.00258
	GO:0010517	regulation of phospholipase activity	2023/2/5	66/18800	0.01093	0.00307
**CC**	GO:0005938	cell cortex	2023/2/5	310/19594	0.05377	0.0259
	GO:0031983	vesicle lumen	2023/2/5	327/19594	0.05377	0.0259
	GO:0031092	platelet alpha granule membrane	2023/1/5	17/19594	0.05377	0.0259
	GO:0005925	focal adhesion	2023/2/5	419/19594	0.05377	0.0259
	GO:0030055	cell-substrate junction	2023/2/5	428/19594	0.05377	0.0259
	GO:0015629	actin cytoskeleton	2023/2/5	499/19594	0.06048	0.02914
	GO:0005771	multivesicular body	2023/1/5	63/19594	0.08852	0.04264
**MF**	GO:0003779	actin binding	2023/3/5	439/18410	0.01196	0.00287
	GO:0031625	ubiquitin protein ligase binding	2023/2/5	298/18410	0.02641	0.00635
	GO:0004955	prostaglandin receptor activity	2023/1/5	10/18410	0.02641	0.00635
	GO:0044389	ubiquitin-like protein ligase binding	2023/2/5	317/18410	0.02641	0.00635
	GO:0004954	prostanoid receptor activity	2023/1/5	11/18410	0.02641	0.00635
	GO:0008179	adenylate cyclase binding	2023/1/5	12/18410	0.02641	0.00635
	GO:0097677	STAT family protein binding	2023/1/5	12/18410	0.02641	0.00635

GO, Gene Ontology; BP, biological process; CC, cellular component; MF, molecular function.

**Figure 11:**
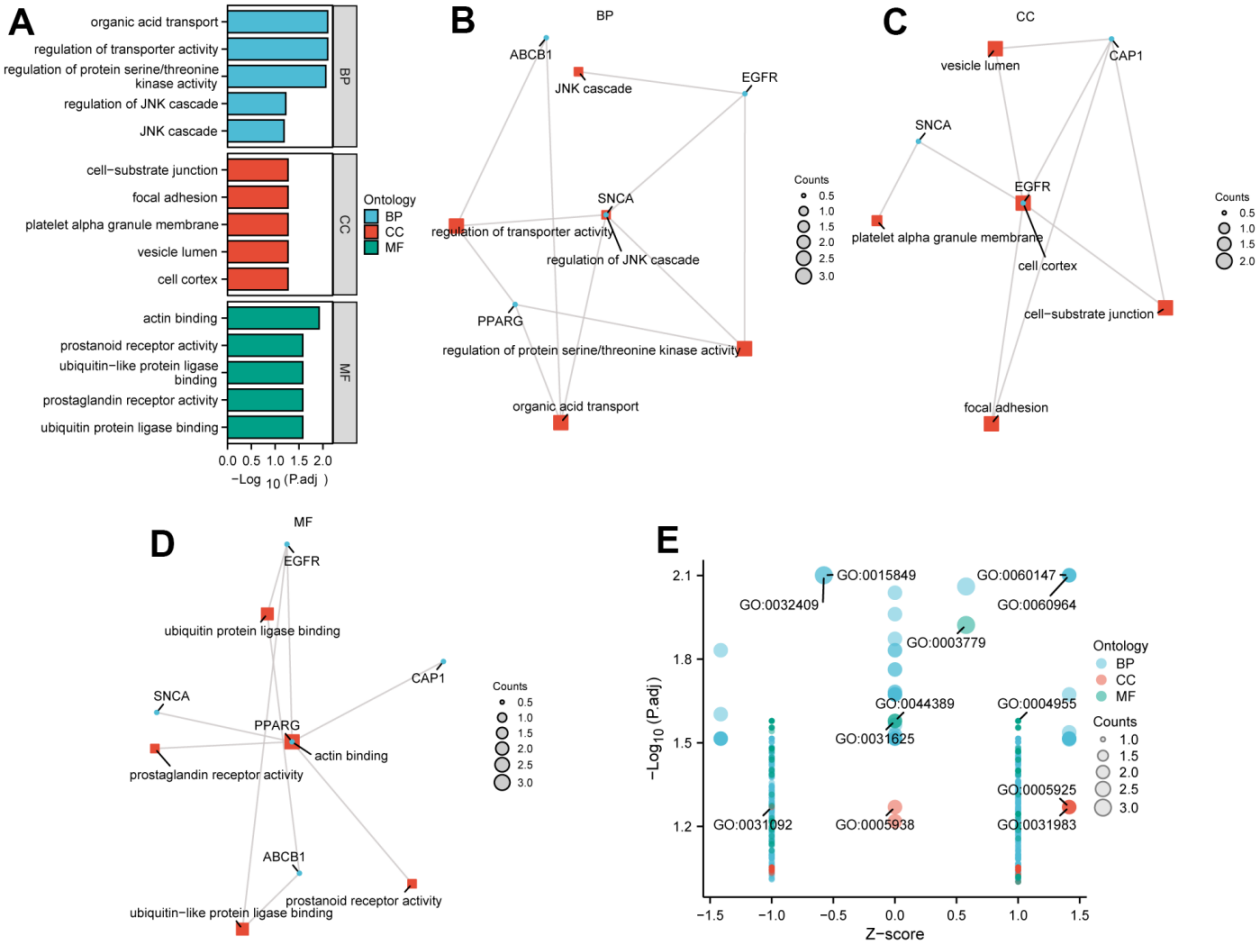
**GO enrichment analysis.** (**A**)The GO enrichment analyses of DEGs in Hub genes. (**B**–**D**) Chordal graph of GO enrichment for 5 Hub genes: BP pathway (**B**), CC pathway (**C**), and MF pathway (**D**). In the network diagram, blue dots represent specific genes, and red blocks represent specific pathways. (**E**) A bubble plot shows GO enrichment of a joint logFC. GO, Gene Ontology.

We presented the results of GO annotations using bar charts (Figure 11A) and network charts (Figure 11B–11D). Subsequently, the GO annotations of a joint logFC in these 5 hub genes were conducted. Based on the enrichment analysis, we calculated the z-score corresponding to each molecule by providing the logFC values of the key genes from the difference analysis results of the TCGA-PAAD high and low-risk groups. We showed the GO enrichment analysis results of a joint logFC using a bubble plot (Figure 11E).

We further performed GSEA between the high and low-risk groups based on the prognostic model to uncover enrichment signaling pathways in PAAD with P.adj < 0.05 and FDR value (qvalue) < 0.25 (Supplementary Table 2). The results showed that genes in the high and low-risk groups were significantly associated with NO2IL12 pathway (Supplementary Figure 5B), peptide hormone metabolism (Figure 8C), ADORA2B mediated anti-inflammatory cytokines production (Supplementary Figure 5D), metabolic reprogramming in pancreatic cancer (Supplementary Figure 5E), MET promotes cell motility (Supplementary Figure 5F), assembly of collagen fibrils, and other multimeric structures (Supplementary Figure 5G). We drew a ridge plot (Supplementary Figure 5A) to display the above results.

We also conducted a GSVA to explore the differences of Hallmark genes in the prognosis model between the high and low-risk groups. The results showed significant differences in the high and low-risk groups of the TCGA-PAAD prognostic model among 36 hallmark genes sets, including adipogenesis, androgen response, apical junction, apical surface, and apoptosis (Supplementary Table 3). We also analyzed the differential expression of these 36 HALLMARK pathways between high and low-risk groups in the TCGA-PAAD dataset and drew a heatmap to display the results using pheatmap R packet (Supplementary Figure 6A). According to the results of GSVA, we investigated the differences of 36 HALLMARK pathways between different groups in the TCGA-PAAD dataset using the Mann Whitney U test and displayed the results through grouped bar chart (Supplementary Figure 6B), which revealed that the expression of these 36 HALLMARK pathways were significantly different between high and low-risk groups.

### Construction of PPI network and identification of prognostic-related gene

**Figure 12:**
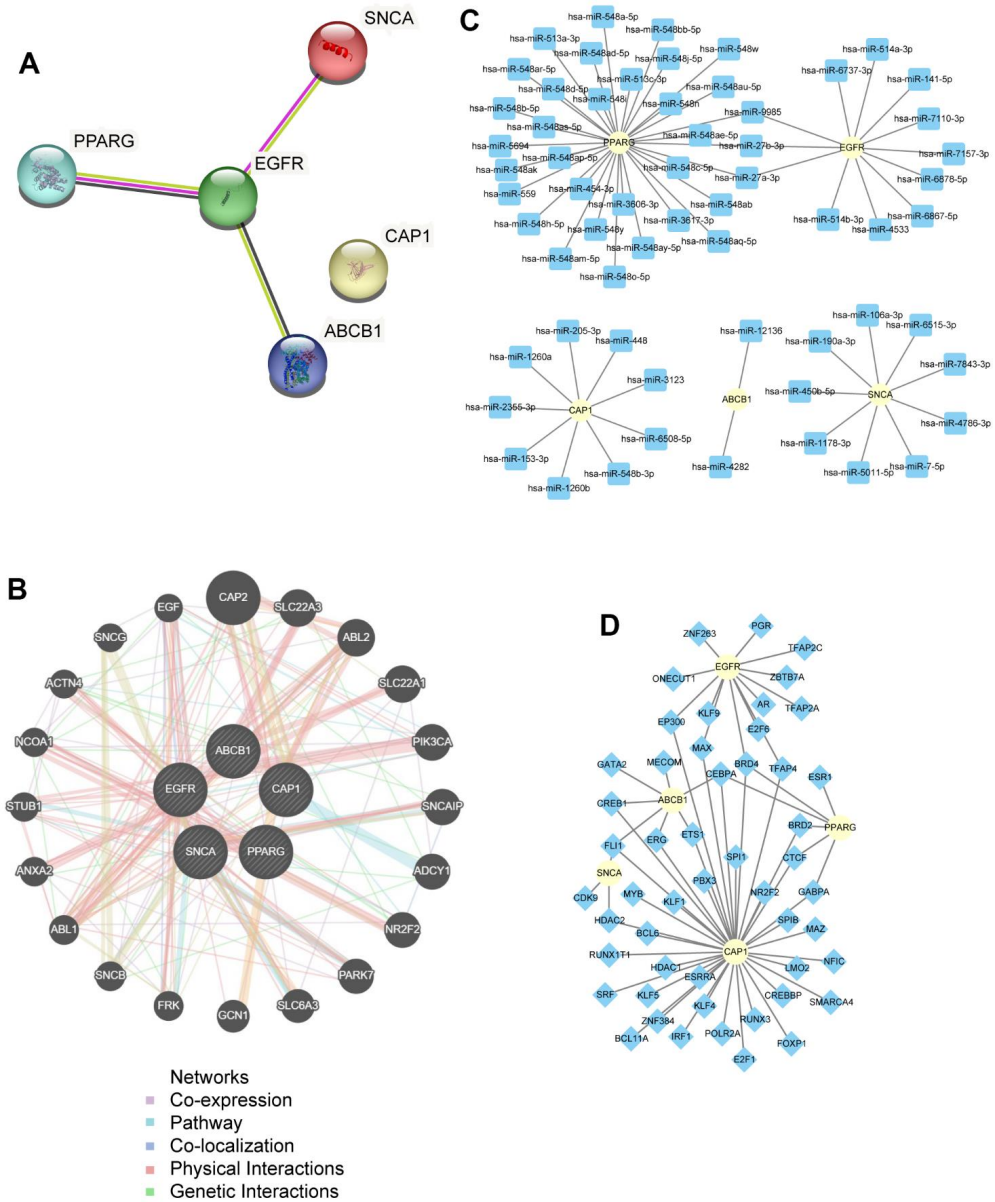
**PPI network analysis.** (**A**) PPI network of hub genes. (**B**) PPI network of functionally similar genes analysis in Hub genes. Black circles with white slashes represent the input hub genes, and other black circles without white slashes represent predicted functionally similar genes; red lines represent physical interactions among genes, purple lines represent co-expression relationships among genes, yellow lines represent shared protein domains among genes, blue lines represent co-localization relationships among genes, and green lines represent genetic interactions among genes. (**C**) An interaction network of mRNA-miRNA in hub genes. The yellow circle represents mRNA; the blue square represents miRNA. (**D**) An interaction network of mRNA-TFs in hub genes. The yellow circle represents mRNA; the blue diamond shape represents TFs. PPI, protein-protein interaction; TF, Transcription Factor.

To better understand the potential interactions between the 5 hub genes (*ABCB1*, *CAP1*, *EGFR*, *PPARG*, *SNCA*) in the PAAD group, a PPI network was constructed using the STRING database, and the interaction threshold was set at 0.400. The PPI analysis is visualized in Figure 12A. The results showed that other hub genes interacted with at least one hub gene except for *CAP1* under the least required interaction score of 0.400. Among them, *EGFR* had interactions with three hub genes, a gene with many interactions with other genes. In addition, we also predicted and constructed an interaction network of functionally similar genes among these five hub genes using GeneMANIA to observe their interactions, co-expression, co-localization, and other information (Figure 12B).

Besides, we used mRNA-miRNA data from the miRDB database to predict miRNAs that interact with five hub genes. We then screened with the criteria of target score>90, and the interaction pairs were visualized by Cytoscape to show mRNA-miRNA interaction network (Figure 12C). A total of 65 pairs mRNA-miRNA interactions were constituted by 5 hub genes (*ABCB1*, *CAP1*, *EGFR*, *PPARG*, *SNCA*) and 62 miRNAs (Supplementary Table 4).

TF control gene expression through interactions with target genes (mRNA) during the post transcriptional stage. We searched for TFs combined with hub genes through CHIPBase and hTFtarget database, downloaded the interaction relationships found in the two databases, and performed the intersection. Finally, a total of 67 pairs of mRNA-TFs interaction relationships were constituted by 5 hub genes (*ABCB1*, *CAP1*, *EGFR*, *PPARG*, *SNCA*) and 51 TFs, which were visualized using Cytoscape (Figure 12C). The specific mRNA-TFs interaction relationships are shown in the Supplementary Table 5.

## DISCUSSION

Due to the presence of atypical symptoms, insidious location, rapid disease progression, and poor prognosis, PAAD has emerged as one of the most malignant and aggressive solid tumors [32]. Although surgical and adjuvant treatment for pancreatic cancer has been extensively developed, it is still insufficient to improve the prognosis of PAAD patients. However, exploration and mining of effective biomarkers in pancreatic cancer have become increasingly vital and beneficial for diagnosing, treating, and supervising PAAD patients, which are still relatively scarce. Serum CA19-9 is the only serum biomarker for treatment approved by the Food and Drug Administration [33], and it also has a certain suggestive effect on the evaluation of prognosis and overall survival rate in PAAD patients [34]. Nevertheless, CA19-9 is significantly elevated in a variety of benign diseases of the digestive system, such as cirrhosis, pancreatitis, and cholangitis. Furthermore, there are non-specific expressions of false negative results caused by the Lewis negative genotype and false positive results caused by obstructive jaundice, which severely limits the application of serum CA19-9 in the PAAD prognosis prediction [35]. Thus, it is crucial to identify novel biomarkers to predict prognosis and enhance individualized therapies in PAAD patients. Exosomes, as novel biomarkers for tumors, have been developed for constructing prognostic models in esophageal carcinoma [36], hepatocellular carcinoma [37], and pancreatic ductal adenocarcinoma [38]. Nowadays, increasing studies have found that abnormal lipid metabolism is associated with tumors, and researchers also constructed a prognostic model based on lipid metabolism-related genes to predict patients with gastric [39] and hepatocellular carcinoma [40]. Even though exosomes or lipid metabolism genes have been shown to be accurate predictors of tumor prognosis in previous studies, there is still lack of research on EALMRGs as a predictive biomarker for PAAD.

In this study, we constructed a 7-gene (*ABCB1*, *CAP1*, *EGFR*, *ITGB1*, *MAPK1*, *PPARG*, *SNCA*) prognostic model based on EALMRGs using LASSO logistic regression analysis. Calibration analysis results suggested that the prediction performance in 1- and 3-year were better than that in 5-year; however, the DCA analysis results were the opposite. Several previous studies also reported some specific prognostic models in PAAD. For example, Yang et al. constructed a 3-gene (*ALOX5*, *ALOX12*, *CISD1*) prognostic model based on ferroptosis-related genes, and they found that the model had a high AUC (=0.976) at 5-year in TCGA-training set, as well as in the GSE62452 (AUC=0.743) [41]. Moreover, a novel 9-gene prognostic model based on multi-omics in PAAD was developed and proven to have a good performance (average AUC>0.8) in predicting the prognosis [42]. Furthermore, Su et al. reported the high prognostic performance of pyroptosis-related genes (*GSDMC*, *IRF1*, and *PLCG1*). They found that this model performed well in predicting the 1-, 3- and 5-year survival of TCGA-PAAD patients [43]. Despite this, our model demonstrates good predictive value in general, and we are the first to examine EALMRG signatures in PAAD prognosis prediction.

We also obtained 13270 DEGs between high and low-risk groups based on the EALMRGs related prognostic model from TCGA-PAAD, with 8016 up-regulated genes and 5254 down-regulated genes. The GSEA results showed that these DEGs were closely related to metabolic reprogramming in pancreatic cancer. Metabolic reprogramming in pancreatic cancer, including glucose metabolism, lipid metabolism, tricarboxylic acid cycle, and amino acid metabolism, not only provides nutrition for tumor occurrence and development, but also affects the function of anti-tumor immune cells and immunosuppressive cells in the microenvironment [44]. Besides, mesenchymal-epithelial transition (MET) promoting cell motility plays an important role in various tumors [45]. However, the GSVA results suggested that these DEGs differed in 36 Hallmarks, such as androgen response and apical junction. A previous study reported that androgen receptors and related responses were associated with human carcinogenesis in hepatocellular and pancreatic cancer [46]. The androgen response has also been proven to play a vital role in the occurrence and development of tumors [47]. The results of our study were consistent with this literature, but the specific mechanism in PAAD still needs further exploration.

We identified 5 significant EALMRGs, including *ABCB1*, *CAP1*, *EGFR*, *PPARG,* and *SNCA*. The results suggested that all of them have good predictive performance in the fifth year, with *ABCB1* and *SNCA* as protective factors having better overall predictive ability at 1-, 3- and 5-year. These 5 genes are closely distributed on chromosomes, indicating a close functional connection at the genomic level. Thus, we further explored the biological role of these 5 genes and PAAD. Consistent with the published data, these 5 genes were enriched in several GO terms, such as protein serine/threonine kinase activity [48], focal adhesion [49], and actin binding [50], which have suggested the possible relationship with their regulation and mediation in PAAD patients. The enrichment analysis result also indicated that cell-substrate junction is involved in the progress of several cancers, including gastric [51], nasopharyngeal [52], and lung cancers [53], but pancreatic cancer has not yet been reported. However, disease and disorder research on cancers has not been conducted in relation to the organic acid transport and regulation of transporter activity, which is likely a direction for our research on tumor mechanisms.

Through PPI construction, we identified several genes, including mRNA (*CAP2*, *SLC22A3*), miRNA (*miR-9985*, *miR-27*, *miR-548*), and TFs (*CEBPA*), involved in the prognosis mechanism of pancreatic cancer. The *SLC22A3* and *CAP2* might become specific predictive signatures for diagnosing pancreatic cancer [54–56]. *Has-miR-9985-584p* could regulate most identified ferroptosis-related genes to participate in the developing type 2 diabetic islets [57], which is still an important pathogenic factor for pancreatic cancer. MicroRNAs such as *hsa-miR-27a-3p* and *hsa-miR-27b-3p* were found to correlate with *EGFR* and *PPAG* in PAAD, and these 2 microRNAs were also correlated with gastric and esophageal cancers [58, 59]. However, *hsa-miR-548-family* seems to embrace a very close relationship with *PPARG* from our study, and it belongs to the top down-regulated colorectal cancer from Josef Horak’s research [60]. Dysregulation of *CEBPA* expression is widely reported in human cancer, for which various mechanisms have been described [61]. A study reported that *CEBPA* could promote the proliferation, invasion and migration of pancreatic cancer cells, and upregulation of *CEBPA* can be induced by *KDM6B* knockdown [62]. The above results not only indicate that these 5 EALMRGs have great potential to predict the prognosis of PAAD forebode the possible interaction of genes and molecules, and further provide a theoretical basis for the EALMRGs prognosis mechanism.

Currently, an increasing body of evidence suggests that these 5 hub genes are associated with the malignancy of tumors, especially PAAD. ATP-binding cassette transporter B1 (*ABCB1*) is called MDR1 P-glycoprotein and has been reported to play an important role in the chemotherapy resistance of pancreatic cells through upregulation of drug efflux [63]. *ABCB1* is a pivotal transcriptional factor of WNT/β-catenin signaling, and the upregulation of ABCB1 expression was modulated by the specific gain-of-function *CTNNB1* mutations [64]. Notably, transcriptional level of *ABCB1* is also increased through leptin activation from tumor-related microenvironment [65]. Another research reported that patients with high protein levels of *ABCB1* are correlated with worse prognosis [66]. However, our study found that an up-regulated level of *ABCB1* could improve the survival rate of pancreatic cancer. The opposite result might relate to the association of *ABCB1* with molecular status, tumor characteristics, demographics, and genetic variants [67], which still needs more laboratory and clinical data to be explored and validated.

Initially, *SNCA* was firstly identified as a pivotal promoter in the development of Parkinson’s disease [68] and is important in maintaining mitochondrial homeostasis, proteasome function, and molecular chaperone activity [69]. Several studies reported that *SNCA* is related to carcinogenesis. In a meta-analysis of genetic parkinsonism and cancer, SNCA was predominantly associated with gastrointestinal cancers [70], such as colorectal cancer [71]. Matteo Bianchini and co-workers discovered that α-syn was significantly upregulated in PDAC [72]. Nevertheless, our study found that SNCA was positively associated with the survival rate of PAAD patients, suggesting that an intricate molecular mechanism exists between SNCA and PAAD that needs to be further explored.

CAP1 has been reported with upregulation in multiple gastrointestinal, breast, and lung cancers [73–84]. Peroxisome proliferator-activated receptor gamma (*PPARG*) is a member of the nuclear receptor superfamily of ligand-activated transcription factors [85], which is involved in lipid and glucose homeostasis and adipocyte inflammation and differentiation, among other activities [86–88]. Furthermore, many studies have shown that *PPARG* plays a vital role in regulating the growth of several different cancers, including prostate [89], bladder [90], breast [91], and colorectal cancer [92]. The *PPARG* and *DNMTs* appear interrelated in pancreatic cancer, and this interaction might influence cell phenotype and disease behavior [93]. Together with our study, these results suggest that *PPARG* embraces potential value in predicting the prognosis of PAAD patients, remaining to be further explored.

*EGFR* belongs to a family of receptor tyrosine kinases that comprises three other members [94–96]. Several studies have revealed that the increased expression of *EGFR* is widely identified in multiple cancers [97–102]. *EGFR* was reported with overexpression in most proportions of PDAC and correlation with multiple clinical characteristics [103, 104]. These studies indicated that *EGFR* might be a potential prognostic gene in PAAD.

These 5 EALMRGs have a role in the biological behavior of pancreatic tumors; in addition, they have exhibited good performance in prognosis efficacy for PAAD patients. To our knowledge, the clinical value of exosome and lipid metabolism-related genes has not been fully elucidated in multiple cancers. Before this study, Ye et al. developed the 4-genes risk formula based on lipid metabolism-related genes [105]. Zhu et al. identified 11-genes risk signature formula related to lipid metabolism [106]. However, these formulas did not extend the potential clinical impact of exosome-related genes. Our current study identified the change of exosome and lipid metabolism-related genes with clinical value, and the 7-gene (ABCB1, CAP1, EGFR, ITGB1, MAPK1, PPARG, SNCA) prognostic formula is a novel and reliable predictive index in pancreatic cancer and other multiple cancers.

Nonetheless, there are inevitable limitations to our research, which will be rectified and improved in our following study. Firstly, our data originated from previously published datasets, and we performed second data mining and analysis through our own procedure. More thorough and comprehensive investigations are needed prior to clinical application. Secondly, we validated our model with some other datasets in GEO datasets, this method cannot substitute for validation using in-house clinical data. Consequently, further rigorous prospective studies are needed to evaluate the feasibility and authenticity of the model in clinical applications. Thirdly, despite the 5 genes showing good performance in predicting the prognosis of PAAD patients, further verification of these genes *in vivo* and *in vitro* is still needed.

## CONCLUSIONS

In conclusion, the EALMRGs prognostic model of PAAD was constructed using LASSO regression. The model was evaluated by internal and external validation and showed good prognostic performance. For the first time, we further identified 5 EALMRGs (*ABCB1*, *CAP1*, *EGFR*, *PPARG,* and *SNCA*) that might become prognostic biomarkers of PAAD, of which *EGFR* exhibited better prognostic efficiency than other genes. Additionally, the risk formula not only showed potential prognostic value in multiple cancers, but also manifested pivotal alterations in immune infiltration and biological pathway in pancreatic cancer. These findings could provide an effective and reliable method of predicting the prognosis of PAAD patients, which might be a potential and specific direction for further research.

## Supplementary Material

Supplementary Figures

Supplementary Tables
