# Supplementary material for: Exosome and lipid metabolism-related genes in pancreatic adenocarcinoma: a prognosis analysis
Source: Aging (Albany NY). 2023 Oct 18;15(20):11331–68. doi: 10.18632/aging.205130 (PMC10637811; doi:10.18632/aging.205130)
Supplement: Supplementary Figures [file aging-15-205130-s001.pdf]

SUPPLEMENTARY FIGURES

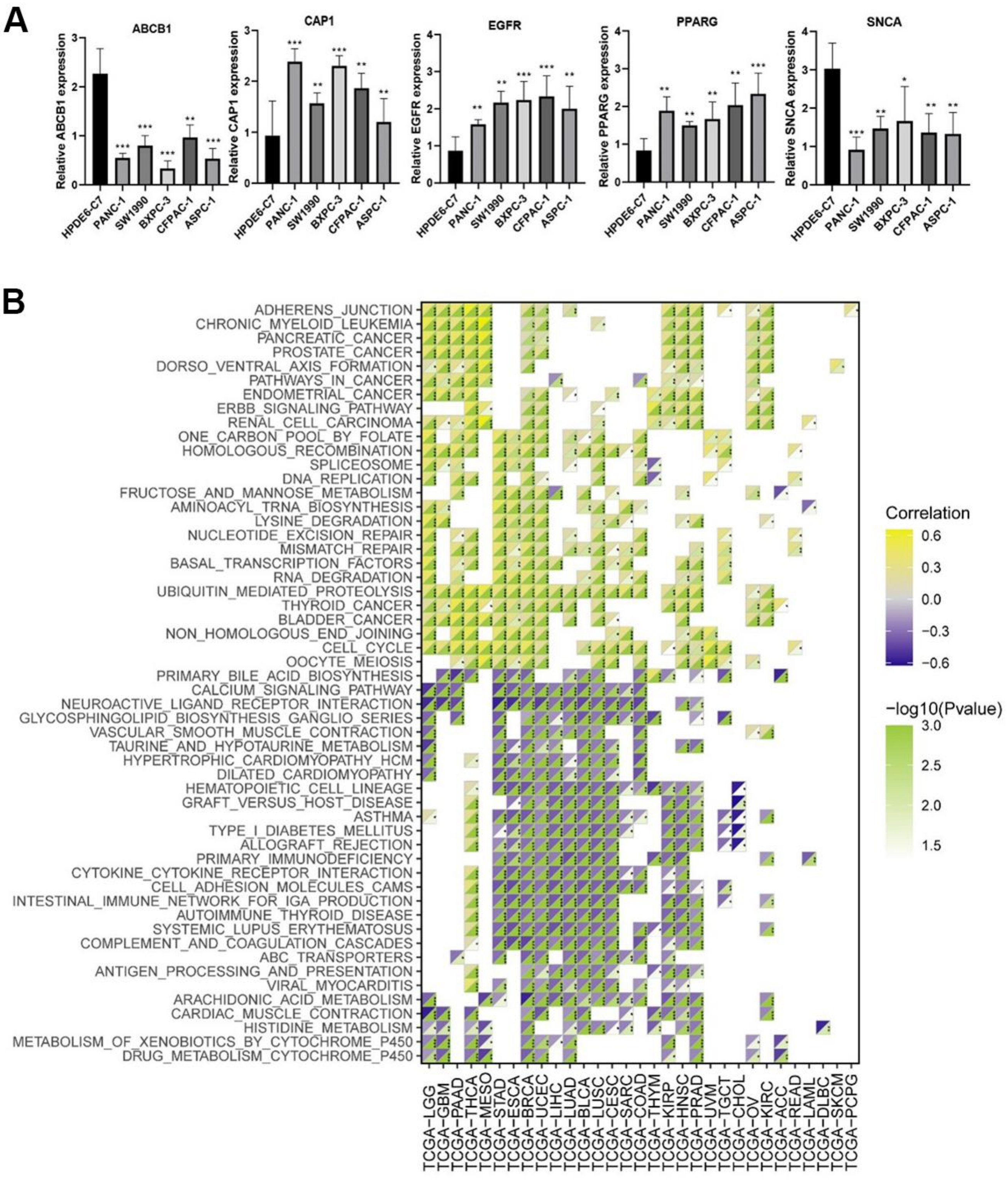

Supplementary Figure 1. (**A**) The expression of the 5 hub genes in pancreatic cancer cell lines comparing to pancreatic normal cell lines. ** means P-value <0.01, *** means P-value <0.001. (**B**) Correlation analysis of GSVA enrichment score and identified risk score in pan-cancer.

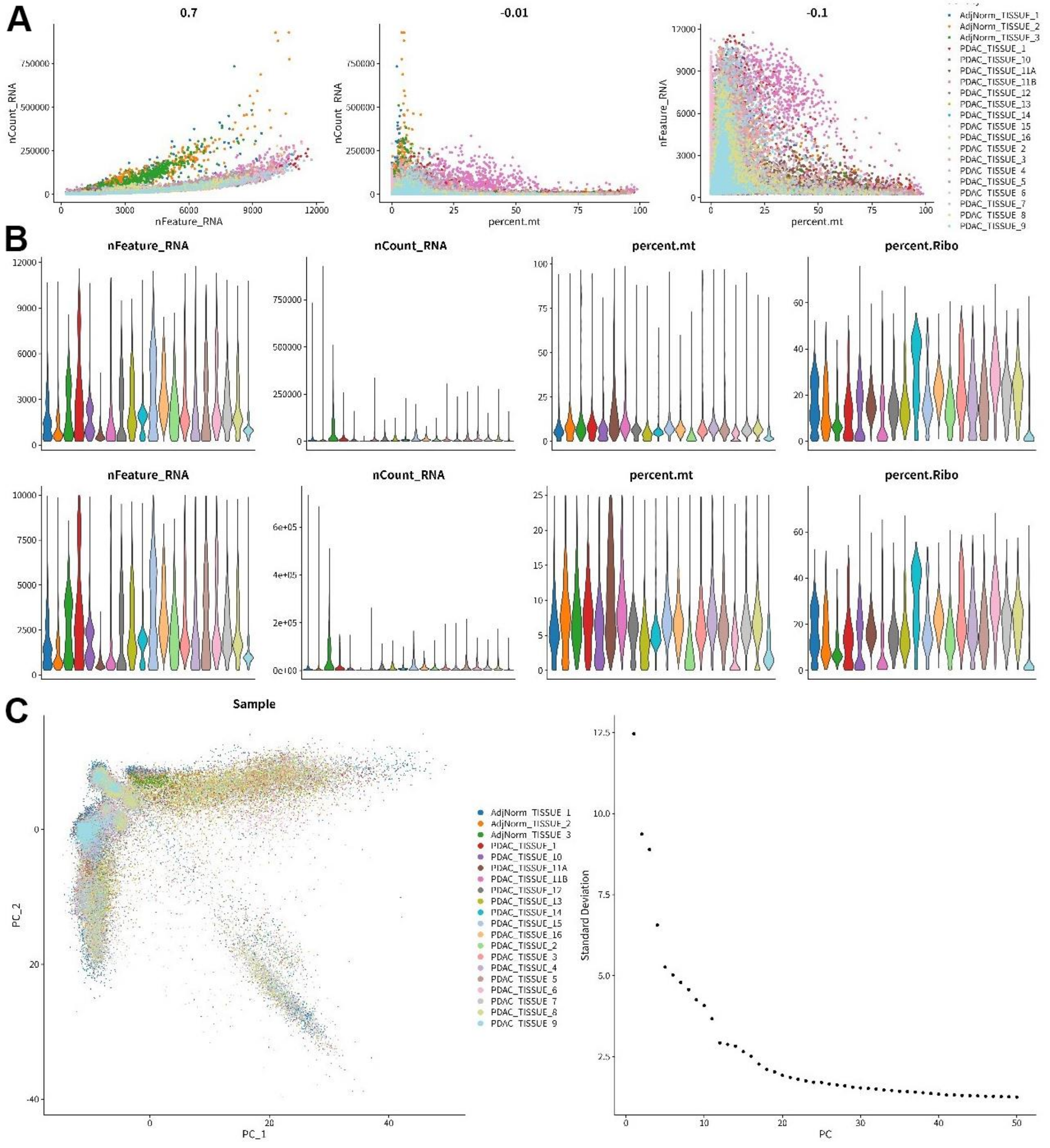

Supplementary Figure 2. (**A**–**C**) The quality of the enrolled single-cell sequencing data.

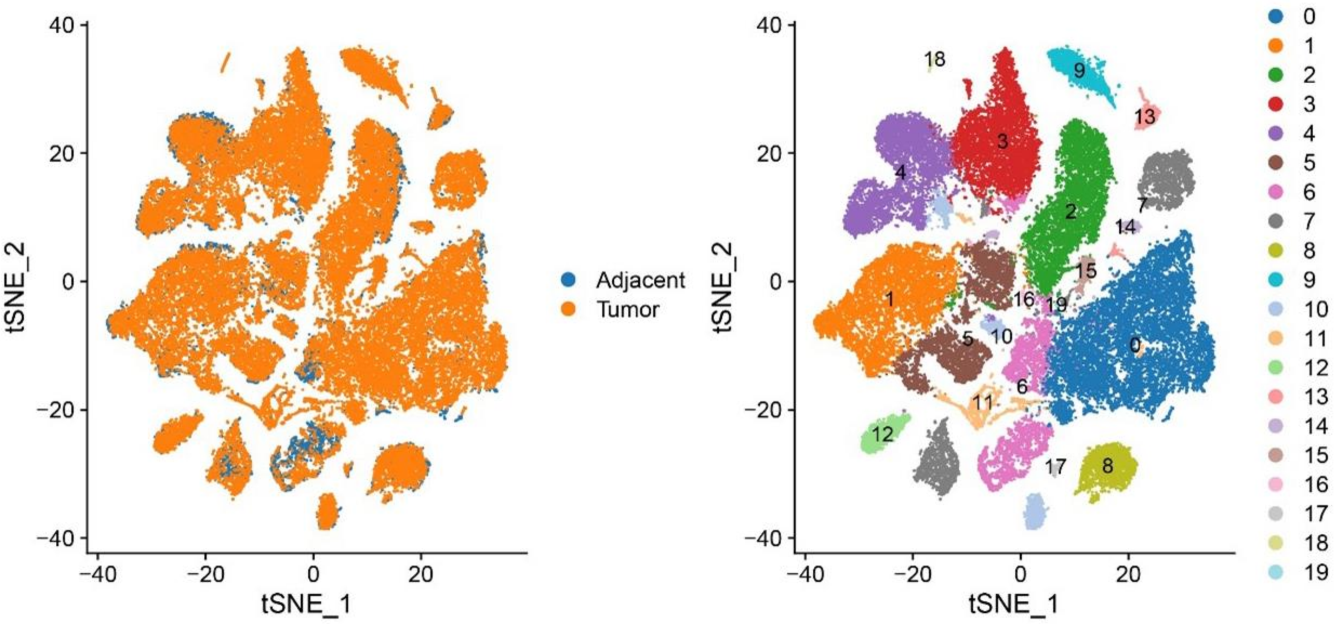

Supplementary Figure 3. Left: The tSNE plot between tumor and adjacent tumor. Right: The tSNE plot of 19 group after clustered.

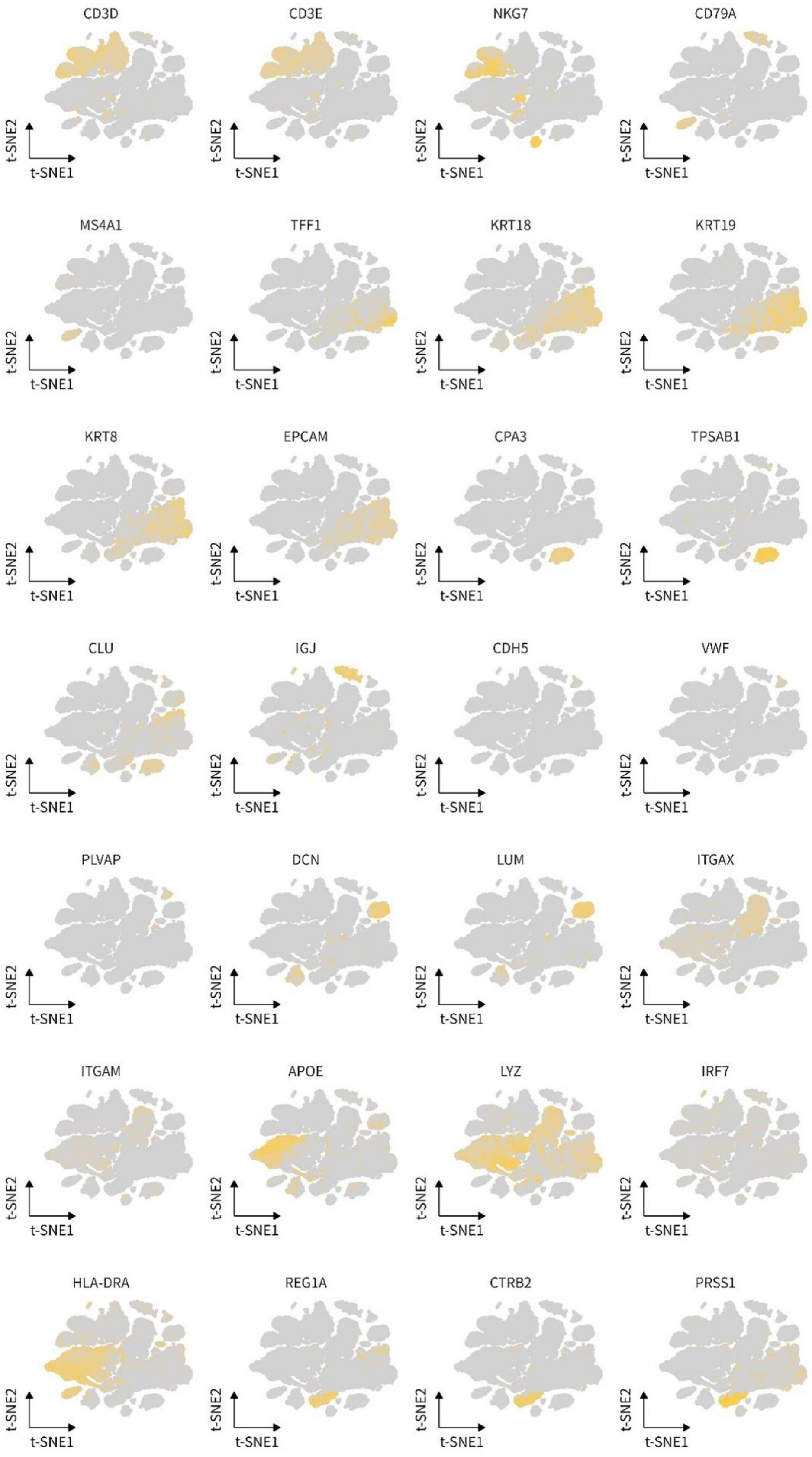

Supplementary Figure 4. The t-SNE plot of multiple markers in single-cell sequencing data.

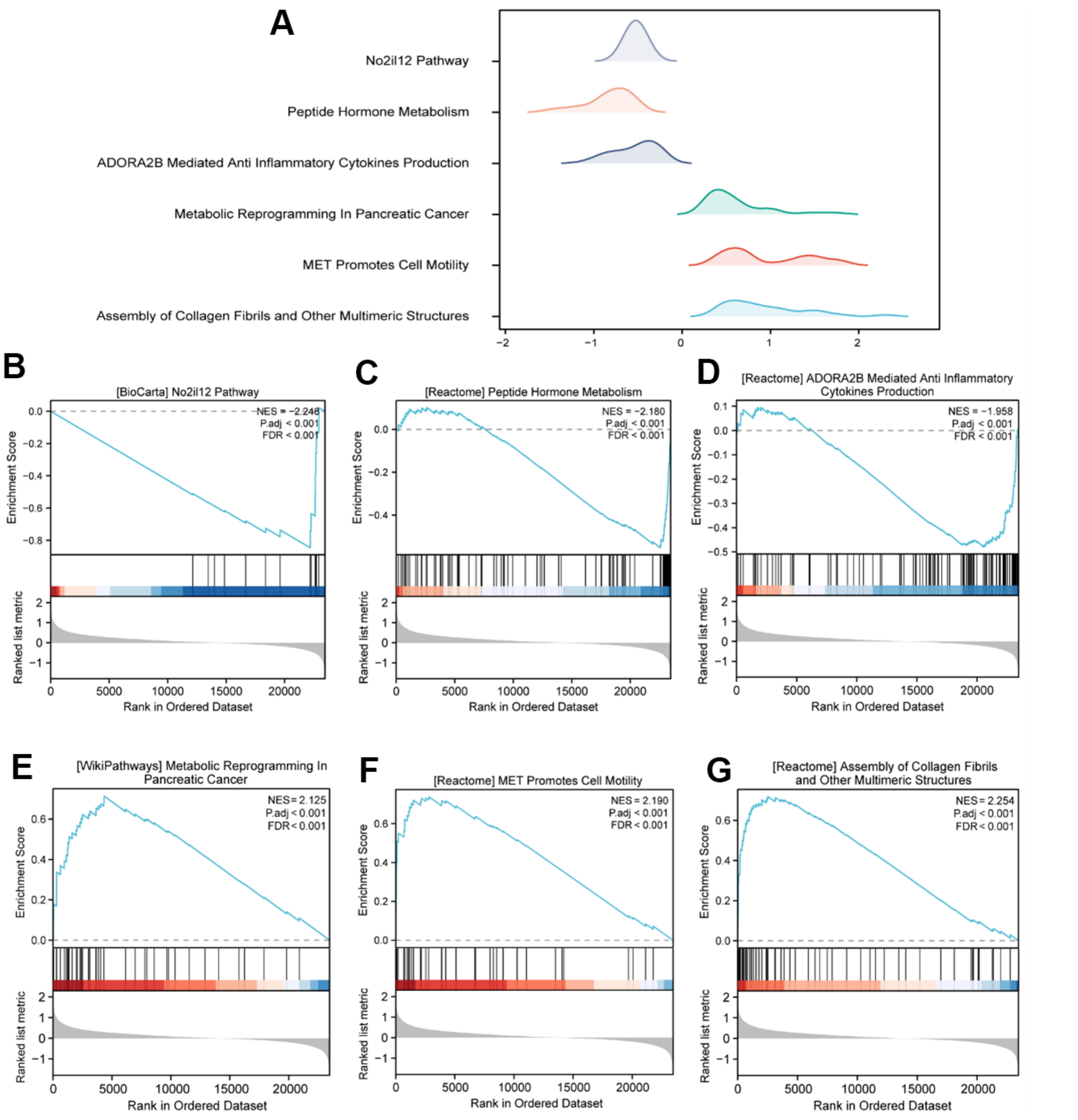

Supplementary Figure 5. (**A**) The identified GSEA pathways between the high and low-risk group. (**B**–**G**) The enrichment score of identified GSEA pathway.

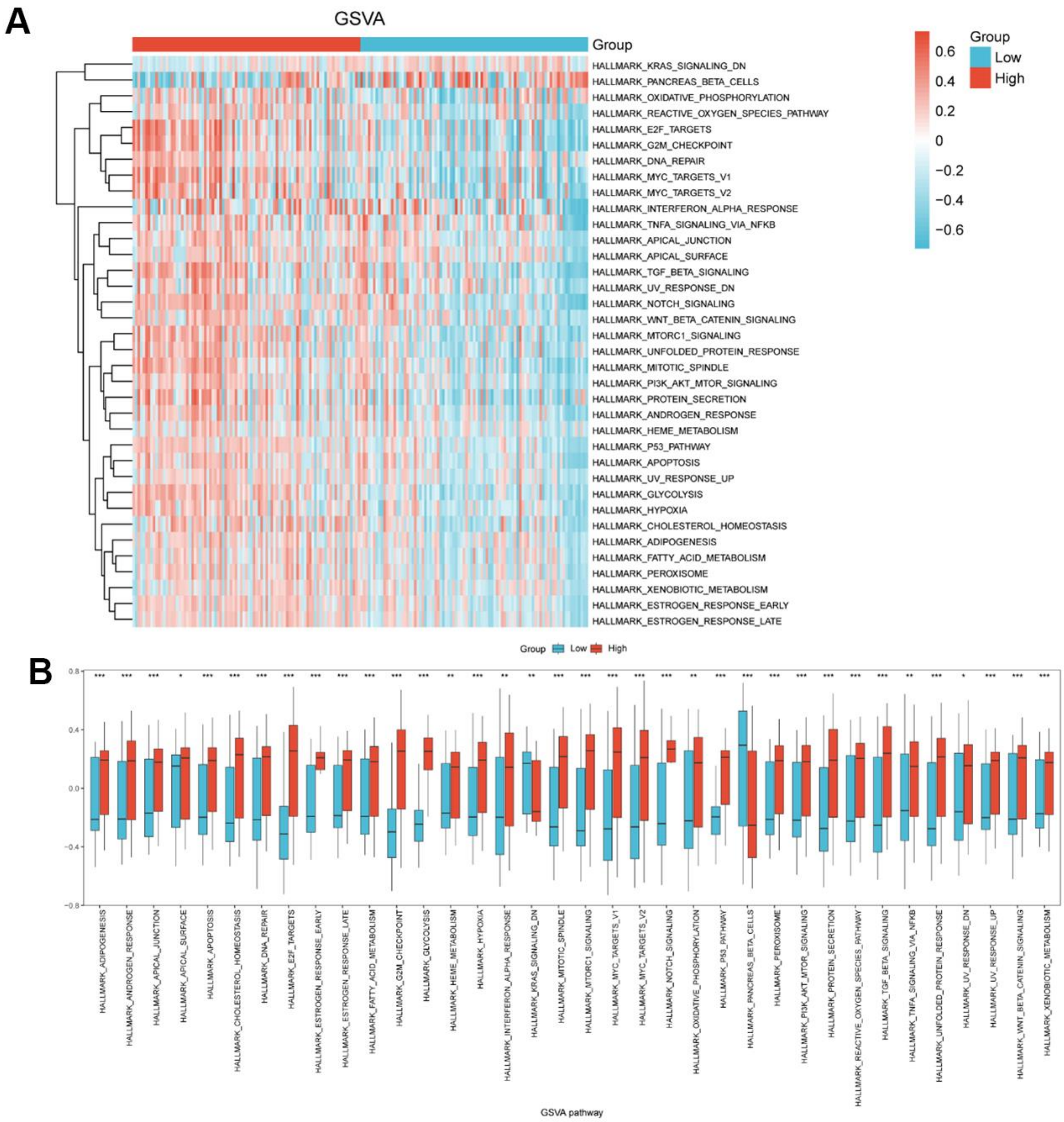

Supplementary Figure 6. (**A**) The identified GSVA pathways in the high and low-risk groups. (**B**) The identified GSEA pathways in the high and low-risk groups.
