# Supplementary material for: Exosome and lipid metabolism-related genes in pancreatic adenocarcinoma: a prognosis analysis
Source: Aging (Albany NY). 2023 Oct 18;15(20):11331–68. doi: 10.18632/aging.205130 (PMC10637811; doi:10.18632/aging.205130)
Supplement: Supplementary Tables [file aging-15-205130-s002.pdf]

SUPPLEMENTARY TABLES

Supplementary Table 1. Exosome and lipid metabolism related genes, EALMRGs.

EALMRGs					
HRAS	*AKT1*	*ACTB*	*ALB*	*CLU*	*FASN*
RAF1	*EGFR*	*TP53*	*GGT1*	*CCL2*	*ABCB1*
RAB7A	*STAT3*	*IL1B*	*SOD1*	*PPARG*	*ITGB3*
BAG6	*ICAM1*	*TGFB1*	*PRNP*	*TNF*	*ACE*
PTEN	*APP*	*GRB2*	*CTNNB1*	*SIRT1*	*IDH1*
TLR4	*ASAH2*	*SNCA*	*HSPA5*	*HSPA1B*	*APOA1*
DPP4	*ITGB1*	*HSPG2*	*VDAC1*	*HIF1A*	*F2*
APOE	*CDC42*	*HSPA1A*	*CAP1*	*VIM*	*NAMPT*
APOA2	*MTREX*	*MAPK1*	*MAPK3*		

Supplementary Table 2. GSEA enrichment analysis results of hub genes.

ID	SetSize	Enrichment score	NES	P-value	P.adj	qvalue
REACTOME_ASSEMBLY_OF_COLLAGEN_FIBRILS_AND_OTHER_MULTIMERIC_STRUCTURES	61	0.718364	2.253987	2.31E-10	1.75E-08	9.89E-09
REACTOME_MET_PROMOTES_CELL_MOTILITY	41	0.738372	2.189964	6.95E-08	2.45E-06	1.38E-06
WP_METABOLIC_REPROGRAMMING_IN_PANCREATIC_CANCER	42	0.714381	2.125378	1.14E-06	2.55E-05	1.44E-05
PID_ECADHERIN_STABILIZATION_PATHWAY	41	0.711503	2.110273	7.96E-07	1.86E-05	1.05E-05
REACTOME_SIGNALING_BY_TGF_BETA_RECEPTOR_COMPLEX	92	0.606184	2.026585	1.52E-07	4.70E-06	2.66E-06
WP_AEROBIC_GLYCOLYSIS	11	0.892959	1.968318	7.83E-06	0.000105	5.94E-05
REACTOME_CELLULAR_RESPONSE_TO_HYPOXIA	74	0.599001	1.937214	7.64E-06	0.000104	5.89E-05
REACTOME_TP53_REGULATES_TRANSCRIPTION_OF_CELL_DEATH_GENES	44	0.643168	1.934143	4.23E-05	0.000416	0.000235
REACTOME_REGULATION_OF_MRNA_STABILITY_BY_PROTEINS_THAT_BIND_AU_RICH_ELEMENTS	86	0.586933	1.933052	1.52E-06	3.19E-05	1.80E-05
REACTOME_AUF1_HNRNP_D0_BINDS_AND_DESTABILIZES_MRNA	54	0.622465	1.926354	1.03E-05	0.000129	7.27E-05
REACTOME_METABOLISM_OF_POLYAMINES	58	0.609515	1.905315	1.49E-05	0.000172	9.73E-05
REACTOME_CLASS_B_2_SECRETIN_FAMILY_RECEPTORS	89	-0.36262	-1.43144	0.006947	0.023977	0.013554
BIOCARTA_IL12_PATHWAY	19	-0.56542	-1.59812	0.007225	0.024532	0.013867
WP_SUDDEN_INFANT_DEATH_SYNDROME_SIDS_SUSCEPTIBILITY_PATHWAYS	154	-0.37325	-1.60073	0.000166	0.00126	0.000712
REACTOME_CLASS_C_3_METABOTROPIC_GLUTAMATE_PHEROMONE_RECEPTORS	23	-0.55704	-1.63056	0.007794	0.026038	0.014719
BIOCARTA_IL17_PATHWAY	13	-0.65349	-1.68422	0.013016	0.038538	0.021785
REACTOME_ANTI_INFLAMMATORY_RESPONSE_FAVOURING_LEISHMANIA_PARASITE_INFECTION	204	-0.38274	-1.69503	3.68E-06	5.94E-05	3.36E-05

REACTOME_GLUCAGON_SIGNALING_IN_METABOLIC_REGULATION	32	-0.56229	-1.81894	0.001201	0.006002	0.003393
REACTOME_METABOLISM_OF_ANGIOTENSINOGEN_TO_ANGIOTENSINS	18	-0.68755	-1.90406	0.001362	0.006673	0.003772
REACTOME_INTEGRATION_OF_ENERGY_METABOLISM	107	-0.47311	-1.92199	1.56E-06	3.19E-05	1.80E-05
WP_FATTY_ACID_OMEGAOXIDATION	13	-0.7473	-1.92599	0.000916	0.004851	0.002742
REACTOME_ADORA2B_MEDIATED_ANTI_INFLAMMATORY_CYTOKINES_PRODUCTION	114	-0.4792	-1.95779	5.92E-07	1.48E-05	8.36E-06
REACTOME_PEPTIDE_HORMONE_METABOLISM	89	-0.55222	-2.17985	4.94E-08	1.84E-06	1.04E-06
BIOCARTA_NO2IL12_PATHWAY	15	-0.84594	-2.24776	1.62E-06	3.28E-05	1.86E-05

GSEA, Gene Set Enrichment Analysis.

Supplementary Table 3. Gene set variation analysis.

logFC	AveExpr	t	P-value	P.adj	B
GLYCOLYSIS	0.36444594	-0.01523	9.863187	5.07E-19	2.53E-17
MTORC1_SIGNALING	0.357089084	-0.01789	8.325416	1.19E-14	2.25E-13
NOTCH_SIGNALING	0.347591157	0.004941	8.305719	1.35E-14	2.25E-13
G2M_CHECKPOINT	0.385941475	-0.0264	8.018213	8.12E-14	1.02E-12
MITOTIC_SPINDLE	0.328281017	-0.01179	7.940877	1.31E-13	1.31E-12
P53_PATHWAY	0.262456174	-0.01229	7.614802	9.57E-13	7.97E-12
MYC_TARGETS_V1	0.367066553	-0.02282	7.18631	1.22E-11	8.71E-11
E2F_TARGETS	0.37467454	-0.0379	7.129268	1.70E-11	1.06E-10
ESTROGEN_RESPONSE_EARLY	0.234150989	0.007557	6.531819	5.05E-10	2.80E-09
UNFOLDED_PROTEIN_RESPONSE	0.286734015	-0.02842	6.427516	8.95E-10	4.48E-09
TGF_BETA_SIGNALING	0.294572698	-0.00919	6.17579	3.49E-09	1.59E-08
HYPOXIA	0.241525103	-0.00795	6.123298	4.61E-09	1.92E-08
ESTROGEN_RESPONSE_LATE	0.21123882	0.001622	5.921306	1.33E-08	5.10E-08
CHOLESTEROL_HOMEOSTASIS	0.256458022	-0.0232	5.907857	1.43E-08	5.10E-08
MYC_TARGETS_V2	0.313551213	-0.03854	5.832338	2.11E-08	7.03E-08
PROTEIN_SECRETION	0.285422891	-0.03426	5.619062	6.22E-08	1.87E-07
DNA_REPAIR	0.237067433	-0.01739	5.614501	6.37E-08	1.87E-07
PI3K_AKT_MTOR_SIGNALING	0.223725184	-0.01771	5.53459	9.49E-08	2.63E-07
PEROXISOME	0.200951847	-0.0095	5.209335	4.61E-07	1.21E-06
APOPTOSIS	0.203206043	-0.00695	5.133401	6.60E-07	1.65E-06
UV_RESPONSE_UP	0.182326528	-0.00785	5.016017	1.14E-06	2.72E-06
ANDROGEN_RESPONSE	0.208896672	-0.0156	4.817842	2.82E-06	6.41E-06
APICAL_JUNCTION	0.172333537	0.005362	4.373193	1.95E-05	4.24E-05
ADIPOGENESIS	0.16377501	-0.00324	4.248083	3.27E-05	6.82E-05
FATTY_ACID_METABOLISM	0.167904487	-0.01793	4.233237	3.48E-05	6.96E-05
PANCREAS_BETA_CELLS	-0.254195748	0.008054	-4.19493	4.07E-05	7.82E-05
REACTIVE_OXYGEN_SPECIES_PATHWAY	0.181776847	-0.01654	4.106457	5.81E-05	0.000108
WNT_BETA_CATENIN_SIGNALING	0.163912122	-0.00018	3.825307	0.000173	0.00031
XENOBIOTIC_METABOLISM	0.120265442	0.003998	3.231334	0.001436	0.002476
INTERFERON_ALPHA_RESPONSE	0.178409452	-0.00718	3.183146	0.001684	0.002807
OXIDATIVE_PHOSPHORYLATION	0.152020094	-0.02721	2.923599	0.00385	0.006209
TNFA_SIGNALING_VIA_NFKB	0.13788571	0.004791	2.859715	0.00468	0.007312

HEME_METABOLISM	0.102568627	-0.01779	2.756254	0.006375	0.009659
UV_RESPONSE_DN	0.120433127	-0.00831	2.615587	0.009571	0.014075
KRAS_SIGNALING_DN	-0.088942579	0.016323	-2.53184	0.012098	0.017282
APICAL_SURFACE	0.09332276	0.029442	2.227793	0.026983	0.037476

GSVA, Gene Set Variation Analysis.

Supplementary Table 4. mRNA-miRNA interaction network nodes.

mRNA	miRNA	mRNA	miRNA
ABCB1	*hsa-miR-12136*	*PPARG*	*hsa-miR-548h-5p*
ABCB1	*hsa-miR-4282*	*PPARG*	*hsa-miR-548au-5p*
CAP1	*hsa-miR-1260a*	*PPARG*	*hsa-miR-548d-5p*
CAP1	*hsa-miR-1260b*	*PPARG*	*hsa-miR-548y*
CAP1	*hsa-miR-448*	*PPARG*	*hsa-miR-548ae-5p*
CAP1	*hsa-miR-205-3p*	*PPARG*	*hsa-miR-548ad-5p*
CAP1	*hsa-miR-153-3p*	*PPARG*	*hsa-miR-548aq-5p*
CAP1	*hsa-miR-6508-5p*	*PPARG*	*hsa-miR-548b-5p*
CAP1	*hsa-miR-548b-3p*	*PPARG*	*hsa-miR-548c-5p*
CAP1	*hsa-miR-2355-3p*	*PPARG*	*hsa-miR-548n*
CAP1	*hsa-miR-3123*	*PPARG*	*hsa-miR-559*
EGFR	*hsa-miR-141-5p*	*PPARG*	*hsa-miR-548ak*
EGFR	*hsa-miR-6878-5p*	*PPARG*	*hsa-miR-548bb-5p*
EGFR	*hsa-miR-514a-3p*	*PPARG*	*hsa-miR-548j-5p*
EGFR	*hsa-miR-4533*	*PPARG*	*hsa-miR-3606-3p*
EGFR	*hsa-miR-514b-3p*	*PPARG*	*hsa-miR-9985*
EGFR	*hsa-miR-6867-5p*	*PPARG*	*hsa-miR-27a-3p*
EGFR	*hsa-miR-7157-3p*	*PPARG*	*hsa-miR-513c-3p*
EGFR	*hsa-miR-6737-3p*	*PPARG*	*hsa-miR-27b-3p*
EGFR	*hsa-miR-7110-3p*	*PPARG*	*hsa-miR-513a-3p*
EGFR	*hsa-miR-9985*	*PPARG*	*hsa-miR-3617-3p*
EGFR	*hsa-miR-27a-3p*	*PPARG*	*hsa-miR-5694*
EGFR	*hsa-miR-27b-3p*	*PPARG*	*hsa-miR-454-3p*
PPARG	*hsa-miR-548am-5p*	*PPARG*	*hsa-miR-190a-3p*
PPARG	*hsa-miR-548ay-5p*	*SNCA*	*hsa-miR-5011-5p*
PPARG	*hsa-miR-548o-5p*	*SNCA*	*hsa-miR-6515-3p*
PPARG	*hsa-miR-548ab*	*SNCA*	*hsa-miR-106a-3p*
PPARG	*hsa-miR-548ap-5p*	*SNCA*	*hsa-miR-1178-3p*
PPARG	*hsa-miR-548w*	*SNCA*	*hsa-miR-4786-3p*
PPARG	*hsa-miR-548as-5p*	*SNCA*	*hsa-miR-7-5p*
PPARG	*hsa-miR-548i*	*SNCA*	*hsa-miR-450b-5p*
PPARG	*hsa-miR-548a-5p*	*SNCA*	*hsa-miR-7843-3p*
PPARG	*hsa-miR-548ar-5p*	*SNCA*	

Supplementary Table 5. mRNA-TF interaction network nodes.

mRNA	TF	mRNA	TF
ABCB1	*CEBPA*	*CAP1*	*RUNX3*
ABCB1	*CREB1*	*CAP1*	*SMARCA4*
ABCB1	*ERG*	*CAP1*	*SPI1*
ABCB1	*ETS1*	*CAP1*	*SPIB*
ABCB1	*FLI1*	*CAP1*	*SRF*
ABCB1	*GATA2*	*CAP1*	*TFAP4*
ABCB1	*MECOM*	*CAP1*	*CEBPA*
CAP1	*E2F1*	*CAP1*	*ZNF384*
CAP1	*EP300*	*CAP1*	*CREB1*
CAP1	*ERG*	*CAP1*	*CREBBP*
CAP1	*ESRRA*	*CAP1*	*CTCF*
CAP1	*ETS1*	*EGFR*	*EP300*
CAP1	*FLI1*	*EGFR*	*KLF9*
CAP1	*FOXP1*	*EGFR*	*MAX*
CAP1	*GABPA*	*EGFR*	*ONECUT1*
CAP1	*HDAC1*	*EGFR*	*PGR*
CAP1	*HDAC2*	*EGFR*	*BRD4*
CAP1	*IRF1*	*EGFR*	*TFAP2A*
CAP1	*BCL11A*	*EGFR*	*TFAP2C*
CAP1	*KLF1*	*EGFR*	*TFAP4*
CAP1	*KLF4*	*EGFR*	*ZBTB7A*
CAP1	*KLF5*	*EGFR*	*ZNF263*
CAP1	*BCL6*	*EGFR*	*AR*
CAP1	*LMO2*	*EGFR*	*E2F6*
CAP1	*MAX*	*PPARG*	*GABPA*
CAP1	*MAZ*	*PPARG*	*BRD2*
CAP1	*MYB*	*PPARG*	*BRD4*
CAP1	*NFIC*	*PPARG*	*CEBPA*
CAP1	*BRD2*	*PPARG*	*CTCF*
CAP1	*NR2F2*	*PPARG*	*ESR1*
CAP1	*BRD4*	*SNCA*	*CDK9*
CAP1	*PBX3*	*SNCA*	*FLI1*
CAP1	*POLR2A*	*SNCA*	*HDAC2*
CAP1	*RUNX1T1*		

TF, Transcription Factor.
